# Interpretable reputation driven asynchronous consensus vehicle networking federated learning architecture

**DOI:** 10.1038/s41598-026-53140-z

**Published:** 2026-05-28

**Authors:** Yuanyuan Zi, Yang Zhou

**Affiliations:** 1 School of Cyber Science and Engineering, University of International Relations, No. 12 Poshangcun, Haidian District, Beijing, 100091 China; 2 School of Future Technology, Shanghai University, Shanghai, 200444 China; 3 Water Technology Research Center, Chemical and Biomolecular Engineering Department, Henry Samueli School of Engineering and Applied Science, University of California, Los Angeles, Los Angeles, CA 90095 USA

**Keywords:** Asynchronous federated learning, Interpretable reputation, Directed acyclic graph, Reputation judgments auditable, Engineering, Mathematics and computing

## Abstract

This paper proposes a Vehicle-Road-Cloud-Chain (VRCC) four-layer collaborative framework to address the issues of lack of interpretability, reputation evaluation failure, and architecture centralization vulnerability faced by federated learning in Internet of Vehicles (IoV) under Differential Privacy (DP), Non-Independent and Identically Distributed (Non-IID) data, and Byzantine attacks. The framework achieves explicit decoupling between low-latency data sharing and latency-tolerant collaborative training. Firstly, construct an asynchronous blockchain consensus layer based on Directed Acyclic Graph (DAG), which supports low confirmation latency model interaction record storage in high-concurrency vehicle scenarios; And design a three-layer interpretable reputation evaluation mechanism, integrating historical task performance, Maximum Mean Discrepancy (MMD) Bayesian inference, and task completion contribution, to achieve causal decoupling between “honest high loss” and “malicious low loss reporting” under Differential Privacy noise, and jointly sign and upload it to the chain through the regulatory committee and Roadside Units (RSUs), making reputation judgments auditable and transparent; Further propose a participant selection algorithm based on Deep Deterministic Policy Gradient (DDPG) and reputation partitioning, which synchronously optimizes communication overhead, computation delay, and redundant filtering in dynamic traffic flow, while utilizing local DAG weight-biased random walks to achieve lightweight asynchronous model quality verification. The experiment shows that the cumulative reward of the proposed method can quickly converge and remain stable, verifying its system-level superiority in interpretable robust aggregation and high-concurrency scalability.

## Introduction

Internet of Vehicles (IoV) generate massive spatiotemporal datasets, yet centralized aggregation faces privacy leakage and bandwidth limitations. Federated Learning (FL)^1^ enables decentralized training on edge devices while preserving data sovereignty^2,3^. However, vehicular FL confronts two critical challenges: (1) Non-IID driving data-vehicles operating in distinct traffic contexts produce gradient divergence, yielding locally optimal but globally incompatible models^4,5^; (2) Byzantine attacks where adversaries upload crafted gradients to bias the global model^6–8^. Existing Byzantine-robust aggregation mechanisms, including Krum^9^, coordinate-wise median^10^, and trimmed mean^10^, rely on distance-based heuristics assuming malicious gradients occupy outlier regions in parameter space. These defenses operate as black-box arbiters incapable of distinguishing statistically heterogeneous honest participants from adversarial actors^6,8^. Similarly, attention-based methods^11^ assign learnable weights based on validation loss or gradient similarity, but inherit the same opacity: low attention weights may stem from adversarial behavior or severe non-IID divergence, precluding causal transparency. Sophisticated attackers can craft updates mimicking honest gradient profiles to evade detection, while honest but heterogeneous nodes risk spurious rejection. This lack of interpretability renders such systems unsuitable for safety-critical vehicular applications requiring accountability and forensic traceability^12^. This vulnerability intensifies under Differential Privacy (DP). While DP mechanisms protect against gradient inversion attacks^13–15^, the injected noise obfuscates loss-based quality indicators. An honest vehicle’s local loss may appear artificially high due to DP perturbation rather than poor model quality, whereas adversaries can report artificially low values by computing loss on manipulated data^6,16^. This asymmetry renders MAE/RMSE-based quality assessment fundamentally unreliable in DP-protected vehicular FL, as defenses cannot distinguish noise-corrupted honest nodes from adversaries. Beyond interpretability deficits, centralized parameter servers introduce single points of failure susceptible to attacks, hardware malfunctions, or regulatory seizure^17–19^, and communication bottlenecks as vehicle populations scale^20^. Decentralized alternatives employing gossip protocols^21,22^ or DAG-based consensus either sacrifice convergence guarantees or incur prohibitive overhead under high mobility^23,24^.

To address interpretability deficits, DP-noise vulnerability, and architectural fragility, we propose the Vehicle-Road-Cloud-Chain (VRCC) system.

The main contributions of this paper are summarized as follows:

1. A Non-IID-Resilient Three-Layer Reputation Mechanism with Causal Transparency: We propose a transparent mechanism decoupling trust evaluation into three dimensions: time-decayed historical interactions ($R_1'$), pre-training data compatibility via MMD and Bayesian inference ($R_2$), and posterior model accuracy ($R_3$). Additionally, we leverage Graph Neural Networks (GNNs) to extract feature fingerprints, quantifying topological cognitive consistency^25–27^. Supervised by a regulatory committee and RSUs within a DAG consensus, this mechanism replaces opaque “black-box” defenses, ensuring robust admission control and precise post-hoc auditing under extreme Non-IID conditions.

2) A DAG-Based VRCC Distributed Asynchronous Collaborative Architecture: To overcome single points of failure and communication bottlenecks, we propose an architecture featuring explicit task separation. We implement the chain layer as a local DAG-based blockchain leveraging the Gossip protocol^21,24^ and our GNN-enhanced reputation mechanism. This design supports highly concurrent asynchronous model updates, decoupling low-latency data sharing from collaborative training, and effectively mitigates the high-latency drawbacks of conventional chain-based structures in highly mobile vehicular environments.

## Related work

### Post-hoc black-box dilemma of Byzantine-robust aggregation

Traditional Byzantine defense mechanisms are essentially reactive, geometric distance-based single-round black-box arbitrations. Classical methods such as Krum^9^ achieve robust aggregation by selecting gradients with the minimum Euclidean distance to neighbors, while Trimmed Mean^10^ and coordinate-wise median^10^ filter extreme values through trimming or calculating the median. However, Fang et al.^6^ systematically revealed the vulnerability of this assumption through optimized local model poisoning attacks-carefully designed attacks can evade detection by Krum, Trimmed Mean, and Bulyan^8^. Xie et al.^7^ further proposed an attack strategy based on inner product manipulation, proving that attackers can effectively break existing Byzantine-tolerant Stochastic Gradient Descent (SGD) algorithms through covert gradient tampering, and such attacks are often harder to detect by geometric distance rules in Non-Independent and Identically Distributed (Non-IID) data scenarios. Trust-based defenses attempt to bypass the limitations of geometric aggregation. FLAME^16^ combines HDBSCAN clustering with weight clipping to distinguish Byzantine behavior from Non-IID heterogeneity. FLTrust^28^ leverages a server-side root of trust dataset to calculate trust scores via cosine similarity, demonstrating resilience against high proportions of adaptive attackers. However, these methods mostly employ post-hoc black-box arbitration mechanisms, assigning trust scores without providing causal explanations as to why specific nodes were flagged. Cai et al.^29^ explicitly point out that the primary limitation of existing reputation systems is the lack of integration with system feedback-scores exist but are not embedded into aggregation or incentive mechanisms to form a closed loop. In high-Non-IID Internet of Vehicles (IoV) scenarios, such lagging filtering mechanisms not only incur prohibitive wasted communication overhead but also fail to isolate heterogeneous interference at the source. This motivates us to introduce a pre-training matching mechanism based on Maximum Mean Discrepancy (MMD) and Bayesian inference to proactively screen out nodes with excessive heterogeneity before parameter aggregation.

### Failure of scalar evaluation in dynamic reputation mechanisms

Although stateful dynamic reputation mechanisms compensate for the shortcomings of single-round defenses, existing mechanisms rely excessively on absolute scalar metrics when evaluating tangible contributions to the model. Karimireddy et al.^4^ introduced bucketing mechanisms to mitigate model drift caused by highly heterogeneous datasets, and Tao et al.^5^ instantiated context-aware clustered Federated Learning (FL) for trajectory prediction. However, these strategies do not systematically solve how to perform reliable quality assessment under data heterogeneity. Cai et al.^29^ criticize existing incentive mechanisms for being mostly static and dependent on easily manipulated metrics such as data volume or participation frequency, lacking dynamic feedback for evaluating long-term trustworthiness. An et al.^30^ emphasize that traditional contribution assessment relies too heavily on simple historical interactions and needs to combine multi-dimensional factors such as actual model contribution and data quality. Raza et al.^31^ further note that the dynamic nature of IoV poses severe challenges to traditional trust management-even nodes with high historical reputation may produce low-quality updates in the current task due to sensor failures or network latency, which purely historical systems fail to capture such “unintentional but harmful” behavior. More problematically, existing works still rely heavily on absolute scalar error metrics such as MAE when measuring posterior physical contribution. When Differential privacy noise is introduced or extreme adversarial noise is faced, the injected perturbations inevitably lead to scalar value inflation, causing traditional assessment methods to lose the ability to distinguish “noise-corrupted honest nodes” from “deliberately disguised adversaries.” To break through this assessment blind spot, this paper abandons the traditional paradigm sensitive to absolute values and instead utilizes gradient direction consistency to construct posterior contribution, resisting extreme noise interference.

### Absence of topological interpretability in federated environments

Graph Neural Networks (GNNs) have great potential for modeling topological relationships between distributed nodes, but existing Federated GNNs and interpretability schemes^25–27^ are mostly limited to post-hoc analysis. They fail to be integrated into the model training lifecycle as intrinsic mechanisms and lack closed-loop capabilities to provide real-time causal explanations for trust predictions. This limitation is particularly prominent in the FL framework because post-hoc analysis may require access to private client data. Wu et al.^32^ proposed FedPerGNN for privacy-preserving graph expansion but admitted its framework does not address interpretability. He et al.^33^ introduced SpreadGNN for serverless FL, which similarly lacks a predictive interpretability mechanism. Liu et al.’s^34^ comprehensive survey explicitly identifies interpretability in Federated GNNs as a key unexplored gap. Although GNNs can capture spatial proximity and communication patterns through graph topology, existing frameworks lack mechanisms to explain why specific nodes are assigned low trust scores. To break the black-box dilemma of traditional Byzantine defenses, this paper innovatively extracts the connection weights between GNN nodes as feature fingerprints, quantifying cognitive consistency in the feature space, thereby providing solid topological-level causal transparency support for the Three-layer interpretable reputation evaluation mechanism.

### Consensus and admission bottlenecks in decentralized IoV

Existing decentralized schemes mitigate single points of failure but often decouple the underlying consensus mechanism from high-level model quality assessment, lacking security admission control for highly concurrent vehicular scenarios. Ko et al.^24^ criticize traditional blockchain-FL integration for poor training performance and excessive computational overhead due to complex consensus mechanisms. Zhu et al.^18^ acknowledge that consensus mechanisms introduce latency that grows exponentially with model size. AFL-DAG^35^ provides a solution combining asynchronous FL with DAG blockchain to realize asynchronous model updates to adapt to the high mobility of IoV. However, the underlying consensus weights in such schemes are often not deeply bound to high-level FL training quality, making them susceptible to poisoning by low-quality nodes and lacking security admission control with causal interpretability. To achieve an intrinsic integration of system efficiency and security defense, unlike the aforementioned fragmented strategies, the proposed Vehicle-Road-Cloud-Chain (VRCC) framework deeply embeds the aforementioned Three-layer interpretable reputation evaluation mechanism into DAG verification weights and the PoMR consensus protocol. It constructs a hybrid architecture decoupling computing power and trust, thereby systematically overcoming coupled challenges such as extreme Non-IID heterogeneity, scalar evaluation failure, and black-box arbitration at the underlying consensus level.

## Vehicle-road-cloud-chain system

### System framework

**Fig. 1:**
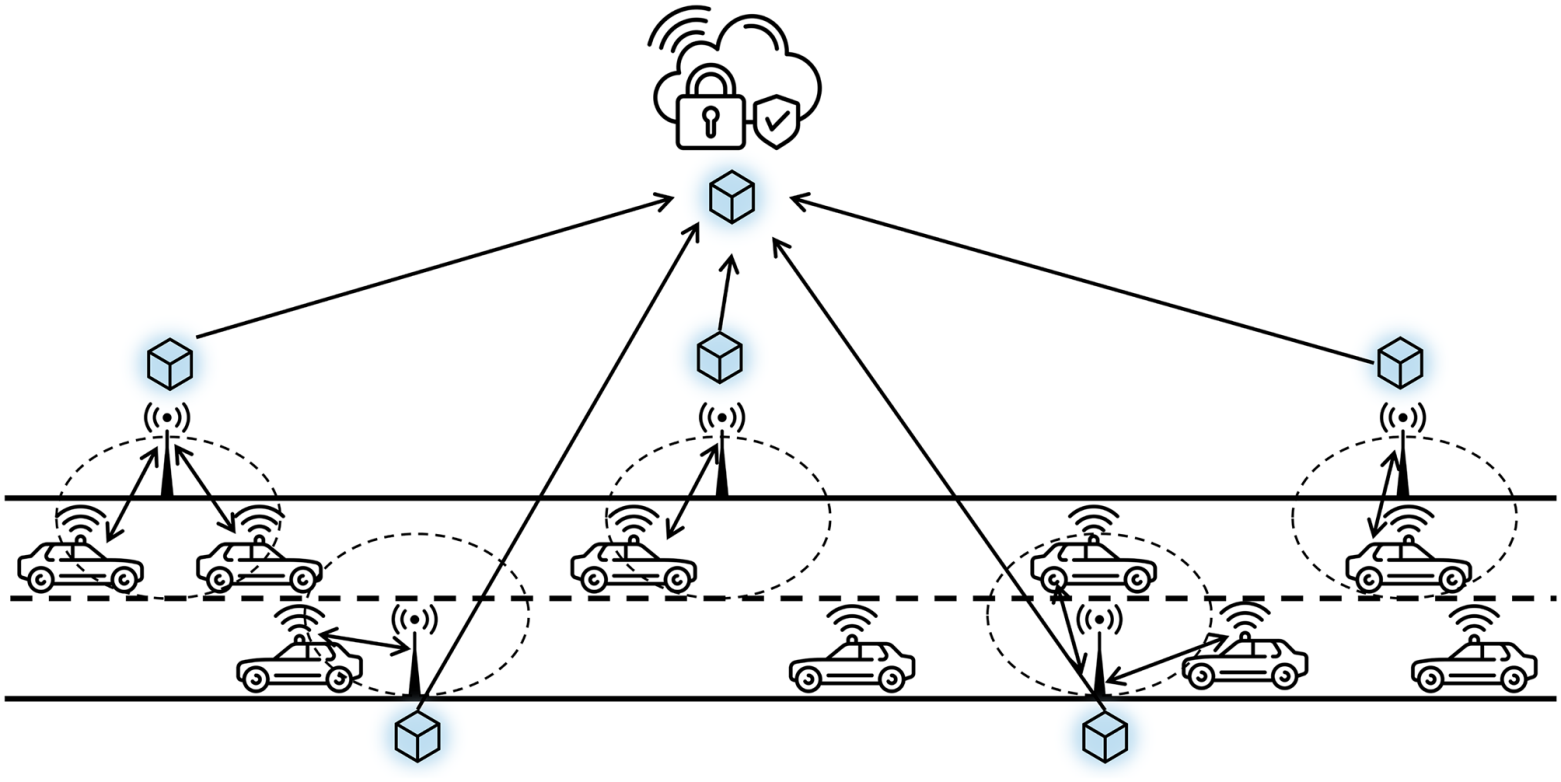
System workflow of the Vehicle-Road-Cloud-Chain framework for data sharing.

In the Internet of Vehicles (IoV), there are various requirements for shared tasks. According to the task delay requirements, the Vehicle-Road-Cloud-Chain system (as shown in Fig. 1) is divided into low-delay data sharing storage tasks and delay-tolerant collaborative sharing training tasks (federated learning). In this context, vehicles $v_i^k$ upload shared task requests to the RSU of the local vehicle cluster, where the RSU of the *k*-th vehicle cluster is denoted as $RS_k$. After verifying the signature through $RS_k$, the shared task request is uploaded to the cloud and the task sharing request list $Req = \{req_1, req_2, \cdots , req_m\}$ is updated in the vehicle blockchain. The execution results are returned to each requesting vehicle, and $Res = \{f(req_1), f(req_2), \cdots , f(req_m)\}$ is updated on the new block.

1 $$\begin{aligned} v_i^k \rightarrow RS_k : req_i = \textrm{Sig}_{SK_{RS_k}}(Type \, || \, Cert_{v_i^k} \, || \, Transaction_{SK_{v_i^k}} \, || \, timestamp) \end{aligned}$$

Among them, according to different request sharing tasks, $Type_1$ and $Type_2$ represent data sharing storage tasks and collaborative sharing training tasks respectively, and generate different transaction records on the blockchain.

2 $$\begin{aligned} Transaction = \{\text {Collaborative sharing}, \text {Data sharing}\} \end{aligned}$$

#### Vehicle-road-cloud-chain networks

**Fig. 2:**
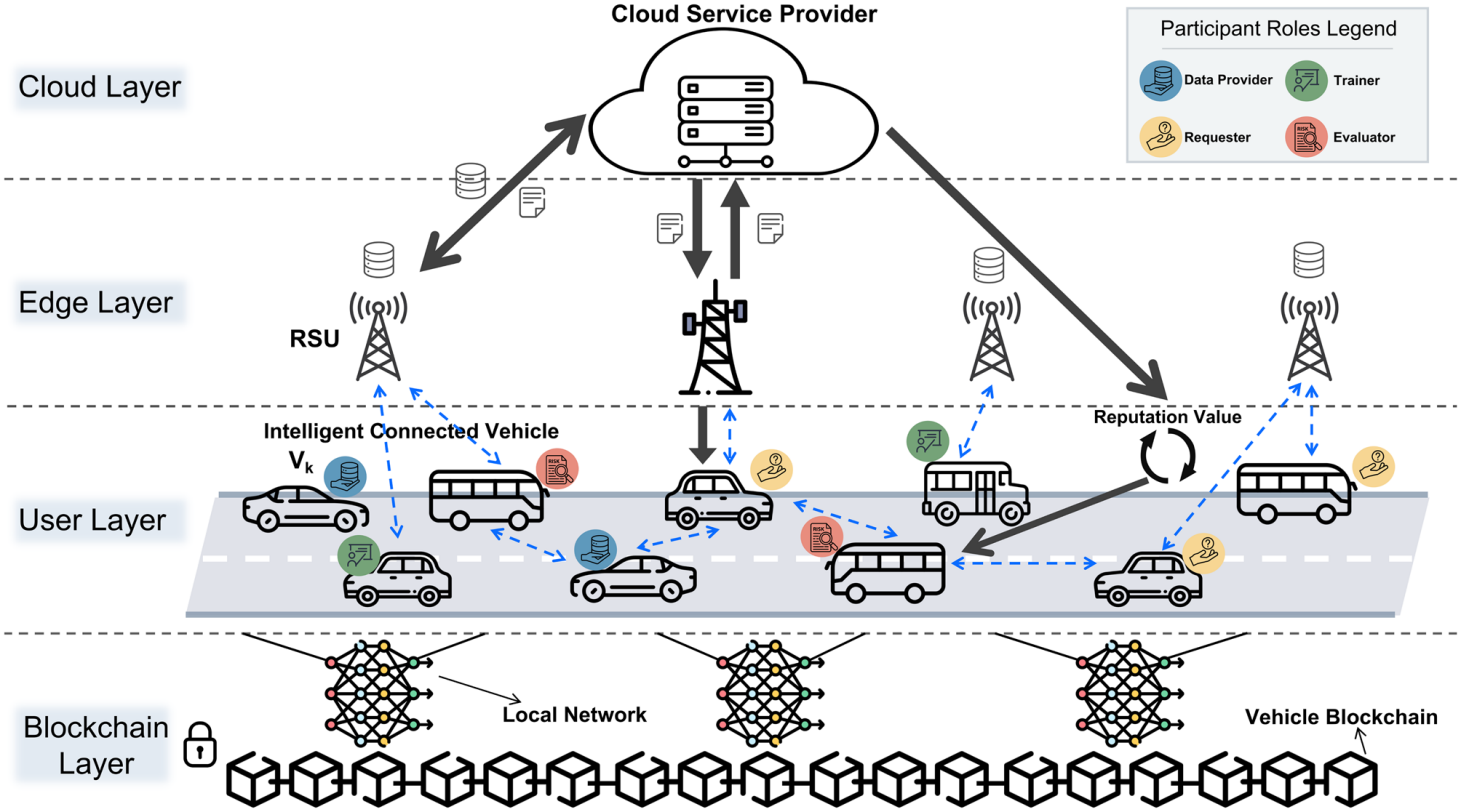
A vehicle-road-cloud-chain system framework.

Our vehicle road cloud chain system is divided into four layers: user layer, edge layer, cloud layer, and blockchain layer (as shown in Fig. 2). The user layer is composed of intelligent connected vehicles. After being authenticated by a trusted authority (such as the government transportation department), the vehicle $v_i$ becomes a legal vehicle and is assigned a unique identity $ID_i$. They obtain real-time environmental information from different dimensions through various sensing devices (lasers, GPS sensors) and can perform local data training tasks. They communicate with nearby RSU through V2R to access local vehicle clusters. Simultaneously, they conduct reputation evaluation and collaborative sharing with other vehicles in the vehicle cluster through V2V interaction. The edge layer is composed of roadside devices (RSU) with certain computing and storage capabilities and a large amount of communication resource capacity. In each area, RSU certified by authoritative institutions compete to become the management node of the vehicle cluster in that area based on their own resources (communication bandwidth and storage capacity). The management node RSU is connected to the cloud layer through upstream communication and connected to the user layer’s CAV through downstream communication. The management node RSU of each vehicle cluster verifies and aggregates the model gradient data recorded on the local DAG node, and uploads the model results and interaction records to the blockchain. The cloud layer is composed of various cloud service providers and has a large amount of computing and storage capabilities. As a regulatory committee node in the vehicle blockchain, it is responsible for storing the entire vehicle blockchain data, verifying and participating in the generation and management of new blocks. At the same time, when performing complex and latency-tolerant computing tasks, the regulatory committee is responsible for synchronous aggregation of the global model and selection of federated learning nodes. For example, implementing iterations of autonomous driving algorithms and updating global high-precision maps.

Our blockchain layer includes vehicle blockchain maintained by RSU and cloud service providers, as well as local networks maintained by various vehicle clusters. All vehicles in each vehicle cluster act as miners in the mining pool and only store local DAG. Vehicle users are also light nodes of the vehicle blockchain, storing the latest block header information. The RSU, which becomes the management node, is responsible for managing local collaborative sharing tasks as the mine owner of the vehicle cluster. It stores the entire blockchain as a full node in the vehicle chain blockchain and ensures the update of local collaborative sharing interaction information on the vehicle blockchain; all management nodes form a management committee in the vehicle blockchain, responsible for recording local vehicle cluster interactions and verifying and signing new blocks. At the same time, they compete for the leadership nodes of the management committee based on their own storage, communication capabilities, and the weighted sum of local vehicle reputation values. The leadership nodes are responsible for generating and updating new blocks in the vehicle blockchain. Cloud service providers are regulatory committee nodes for vehicle blockchain, responsible for auditing, verifying, and signing new blocks. The highly mobile CAV performs partition management based on fixed RSU, and collaborative sharing tasks are assigned to various vehicle clusters for asynchronous federated learning. The management node RSU not only undertakes the local model aggregation iteration of distributed federated learning, but also serves as a consensus node for vehicle blockchain to manage block verification and generation. This means that some nodes can also effectively operate the blockchain. This improves the consensus efficiency of blockchain and achieves the system’s partition resistance.

In the vehicle networking system, there are significant differences in computing and communication resources between vehicles. When vehicle $v_i$ joins the vehicle blockchain network, it is assigned a public key ($PK_i$) and a private key ($SK_i$) based on its unique $ID_i$, and can register different roles in the blockchain network during each collaboration process. At the same time, due to the high mobility of CAV, it cannot be trusted. When a vehicle joins the shared network for the first time, it will be assigned an initial reputation value, and the reputation of the vehicle will be updated based on the execution of each task, and the reputation ledger will be stored in the blockchain. The roles include data provider, trainer, requester, and evaluator. The data provider only participates in data sharing and storage tasks, uploading local data to the management node RSU of the local vehicle cluster. The trainer participates in collaborative sharing tasks, trains the federated learning model using local data, and updates the local DAG with the training results. The requester sends a collaborative sharing request to the management node RSU, which publishes the request record on the vehicle blockchain through a smart contract. The management nodes of each vehicle cluster broadcast it to the local vehicle cluster, and the management committee executes a node selection algorithm to reduce task consumption and improve collaborative security. All CAVs involved in collaborative and shared tasks are evaluators, responsible for assessing the credibility (reputation value) of data providers and trainers. The reputation value of CAVs is aggregated and calculated through the management nodes of the vehicle cluster.

In the vehicle networking system, different types of vehicles generate different types of data, and collecting all vehicle data indiscriminately will only result in a significant waste of storage, transmission, and computing resources. At the same time, the high mobility of vehicles often cannot meet the consistency requirements of distributed data structures for collaborative sharing tasks in the Internet of Vehicles, such as the perception fusion of multiple vehicles on a certain cross road. In order to improve the overall efficiency of collaborative sharing and reduce storage consumption, when each CAV requests to join the local vehicle cluster to assist in sharing tasks from RSU, a transaction record is generated in the vehicle blockchain. Before executing the node selection algorithm for collaborative sharing tasks, the regulatory committee will lock the selected partition for this task based on the transaction information on the vehicle blockchain, which will greatly reduce communication and computational waste. This transaction record includes the identity tag $R_k$ of the local vehicle cluster management node RSU, the unique identity $ID_i$ of the vehicle, available computing resources (CPU cycles/s) $Cp_i$, communication transmission rate $Cm_i$, data type, data distribution $f_i$, historical reputation value, and timestamp. The management node RSU of the local vehicle cluster is responsible for verifying collaborative shared transactions and uploading updated reputation information values of local vehicles to the regulatory committee. The chairman node of the regulatory committee updates the reputation values of all task participants through multi-party searches.

Regulatory Committee: The regulatory committee is composed of various cloud service providers. During each rotation period $T_c$, a cloud service provider is randomly selected as the chairman node, denoted as $Cloud_l$, to manage the global vehicle reputation table update before collaborative sharing tasks and the federated global model iteration in collaborative sharing tasks, while executing node selection algorithms to select participating nodes. The remaining regulatory committee nodes serve as supervisory verification nodes, verifying and signing newly published blocks to ensure efficient consensus of the vehicle blockchain and trustworthy interactions among various vehicle clusters; on the other hand, verifying the updated global vehicle reputation table and node selection algorithm results calculated by the chairman node ensures the credibility of the reputation model of the entire system.

Management Node Committee: The management committee is composed of RSU certified by authoritative institutions for each vehicle cluster. The management node table for the *K*th vehicle cluster is $R_k$. Compared to the high mobility of CAVs in the vehicle cluster, the fixed RSU can serve as a super node to manage and schedule all CAVs in the area. It has been upgraded to have certain computing and storage capabilities as well as high communication bandwidth. The management node has three functions.

Firstly, as the dispatch center of the local vehicle cluster, complete task request verification, update, and broadcasting. After receiving the task request uploaded by the requester, $RS_k$ queries the historical task request of the vehicle blockchain. If the task request is in the historical task, $RS_k$ transmits the result to the requester. Otherwise, it adds the new task request as a transaction record and uploads it to the vehicle blockchain. The current selection node of the regulatory committee processes it and broadcasts it to all nearby vehicles.

Secondly, it is responsible for verifying the updated DAG of the local vehicle cluster, aggregating model parameters, and uploading the global model parameters of the current iteration of the local cluster. The regulatory committee aggregates the model parameters uploaded by all RS participating in the collaborative sharing task to achieve asynchronous model iteration. At the same time, it serves as a consensus node in the vehicle blockchain to verify blocks and compete for block publication.

Finally, the management node also serves as a temporary database for the local vehicle cluster, storing various insensitive raw data collected from vehicles in the area, such as information about construction on roads, traffic conditions, parking lot occupancy rates, and restaurant ratings. These data can be used for various types of collaborative research requests published on the cloud, such as data analysis.

Vehicle cluster: Each vehicle cluster is composed of an RSU at the edge layer as a management node and several CAVs at the user layer. The vehicle table in the *K*th vehicle cluster is $V_k = \{v_1^k, v_2^k, \cdots , v_n^k\}$, and the data shared and collaborated in the Internet of Vehicles is closely related to their geographical location, such as road congestion. Therefore, we divide vehicle clusters according to regions. In the data storage task, vehicles can upload non privacy sensitive data collected in their respective regions to the local data storage RSU to complete iterative updates of the local database. In collaborative shared tasks (federated learning), vehicle clusters utilize local data for multiple rounds of model iteration, which are aggregated by management nodes and uploaded to a regulatory committee chair node for global model updates.

#### Vehicle blockchain architecture

In the Internet of Vehicles, the core requirement for intelligent vehicles is to automatically and instantly control the vehicle to make reasonable movements in complex and unfamiliar environments. The future development direction of the Internet of Vehicles is to shift from rule-based to learning-based perception and decision-making processes. Most existing Internet of Vehicles consider the collaborative sharing of “distributed vehicles and centralized cloud service providers”. CAV uploads local data or model gradients calculated through local data to the cloud, and the centralized cloud synchronously aggregates and updates the global model. A single cloud service provider (cloud) exercises one-way control over user data and abuses data ownership, resulting in users being unable to effectively utilize the value of their data, which will seriously undermine the enthusiasm of vehicle users to participate in collaborative sharing. Therefore, we consider the collaborative sharing market of “distributed vehicles and distributed cloud service providers”. Vehicle users and cloud service providers can choose appropriate collaboration participants based on their task requests. Distributed CAVs can provide perception and planning resources for automotive cloud servers, which can help the former undertake more complex learning tasks and improve model accuracy and robustness. Therefore, combining them in a unified market to achieve mutual reinforcement is a natural and exciting idea.

In our vehicle blockchain system, smart contracts are used to achieve secure, reliable, and efficient collaborative sharing. Smart contracts automatically capture the state values of vehicles on the blockchain through preset rules and trigger operations by receiving trusted external data through an oracle. They cannot be modified or terminated in distributed systems. This system includes reputation retrieval smart contracts (RRSC), collaborative sharing smart contracts (CSSC), and data sharing smart contracts (DSSC) deployed on the vehicle blockchain, as well as user nodes composed of CAV, management committee nodes composed of RSU, and regulatory committee nodes composed of cloud service providers. They jointly maintain the vehicle blockchain to complete secure, trustworthy, and efficient sharing tasks through the PoMR consensus protocol.

On our vehicle blockchain, vehicles initiate task requests to the management nodes of the local vehicle cluster, including applying to participate in collaborative sharing task requests, publishing collaborative sharing task requests, and applying for data sharing task requests. Vehicles send requests to the management nodes of the local vehicle cluster in the form of transactions (as shown in Table 1, Table 2, and Table 3). These interaction records correspond one-to-one with the transaction linked list and are recorded in the block body. At the same time, the hash value of the transaction information is stored in the Merkle tree node and verified to produce new blocks. Therefore, the distributed ledger maintained on our vehicle blockchain is not only used to record interactive transactions, but also to retrieve data, ensuring the trustworthy value transfer of the vehicle road cloud chain system while also improving the efficiency of collaborative sharing tasks in the vehicle blockchain.

**Table 1:** Publish collaborative shared task request transaction records.

Collaborative sharing record	Hash pointer	Requester $ID_j$	Request categories	Information indexes	RSU $R_k$	Timestamp

**Table 2:** Application for participation in collaborative shared task request transaction record.

Retrieval record	Hash pointer	Provide $ID_i$	Data categories	Comm. bandwidth $Cm_i$	Computing resource $Cp_i$	Rsu $R_k$	Reputation opinions $R_1^i$	Timestamp

**Table 3:** Request transaction record for data sharing task.

Data sharing record	Hash pointer	Data owner	Data categories	Data size	Rsu $R_k$	Timestamp

### Three-tier reputation evaluation system

#### Asynchronous evaluation

In the data sharing scenario of the Internet of Vehicles, even vehicles in a cluster may share data unrelated to tasks, which will seriously damage the operation of the entire collaborative sharing market. They may provide incorrect perception information due to the malfunction of perception devices, or they may exaggerate their contributions in shared collaboration or engage in free riding behavior for selfish purposes. The reputation system is an important tool for ensuring user privacy while determining user credibility. The reputation of the target user is calculated by aggregating subjective feedback provided by other users. The level of reputation will affect the likelihood of the user being selected to participate in collaborative shared tasks and the amount of rewards allocated after completing them. Therefore, reputation systems help hold users accountable for their behavior, even if they initially lack trust in the user. In the mobile vehicle nodes of the Internet of Vehicles, we need a multi-layer reputation mechanism maintained by blockchain to ensure trustworthy sharing throughout the entire stage. Under the premise of ensuring data privacy and security as much as possible, the data requester believes that the data provided by the data provider is valid and relevant, and the data provider also believes that their honest performance will receive fair rewards. Therefore, we propose a three-layer reputation asynchronous retrieval mechanism to ensure high-quality collaborative sharing throughout the entire process. Using Bayesian inference to aggregate the maximum mean differences among various vehicle clusters to formulate reputation evaluations generated by vehicle-to-vehicle interactions is a probabilistic information fusion framework based on historical performance and subjective trust in the real world.

**Fig. 3:**
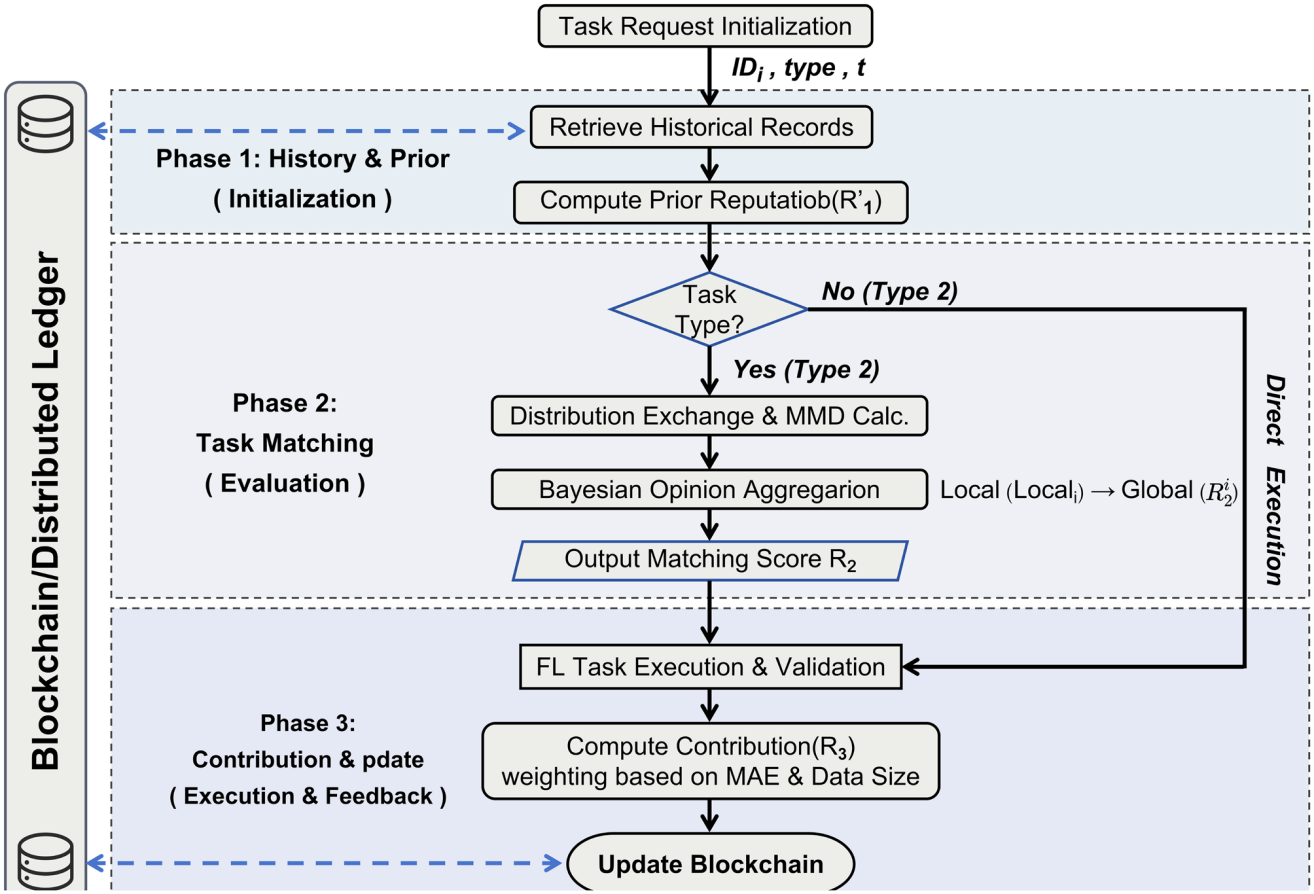
Workflow of the three-tier reputation evaluation mechanism.

How to retrieve the reputation values of participants related to collaborative sharing tasks on the vehicle blockchain is a key issue in the design of this reputation mechanism, as it is necessary to maintain timely updates and retrieval of the reputation values of all vehicles in the distributed system. We have designed a three-layer asynchronous reputation evaluation mechanism. The detailed workflow of the three-tier reputation evaluation mechanism is shown in Fig. 3. Because the reputation value of a vehicle not only reflects its completion status in historical tasks, but also reflects its relevance to the current task. So the reputation of vehicle *i* is composed of three layers of reputation values: historical task performance $R_1$, current task matching degree $R_2$, and task completion contribution $R_3$, forming a reputation vector $Rep_i$ table. Before the task collaboration begins, the super node RSU of each vehicle cluster downloads the initial reputation $R_1$ of the local participating vehicles from the vehicle blockchain, and then aggregates multi-party reputation evaluations to update the reputation information retrieval table of the local vehicles (which includes the vehicle identity *ID*, reputation values $R_1^k$, $R_2^k$, $R_3^k$ of the *k*th task, timestamp, and task number). Finally, the reputation value $Rep_i$ of each vehicle participant after completing the *k*th task will be recorded in the vehicle block in the form of a transaction along with their unique identification *ID*. After being broadcast and verified, it will be added to the vehicle blockchain.

#### Historical task performance (R1)

Consider vehicle $v_i$ applying to the super node RSU of this vehicle cluster to execute a task request and become a task participant (including data provider and trainer), while the task requester is vehicle $v_j$. The initial credibility of task participant $v_i$ towards task requester $v_j$ is determined based on its historical task completion status, as shown in table $R_1$. $R_1 = f_1(x_1, x_2, x_3)$, where $x_1, x_2, x_3$ respectively represent the number of times task completion has been excellent, qualified, and unqualified in the history of vehicle $v_i$. Subtract the actual contribution calculated after each task and the expected contribution before the task starts to obtain the performance of the task completion. The table represents *Rep*. When $Rep> \alpha _0> 0$, the performance of the task completion is excellent. When $-\alpha _0< Rep < \alpha _0$, the task completion is qualified. When $Rep< -\alpha _0 < 0$, the task completion is unqualified. $\alpha _0$ is the measurement coefficient of the performance standard. Here:

3 $$\begin{aligned} \begin{aligned} R_1&= f_1(x_1, x_2, x_3) = R_0 + \alpha _1 x_1 + \alpha _2 x_2 + \alpha _3 x_3 \\ \alpha _1 + \alpha _2 + \alpha _3&= 1, \quad \alpha _1, \alpha _2> 0, \quad \alpha _3 < 0 \end{aligned} \end{aligned}$$

$\alpha _1, \alpha _2, \alpha _3$ are adjustable coefficients, and $R_0$ is the initial value provided to the vehicle for the first collaborative sharing task. The super node RSU of the vehicle cluster to which $v_i$ belongs obtains the latest initial reputation value $R_1$ from the vehicle blockchain, and then obtains $R_1'$ based on $R_1$, which represents the true credibility of task participant $v_i$ before the start of the current task. This is because the initial reputation value $R_1$ obtained solely based on the completion of historical tasks cannot serve as a good prior reputation value for the credibility of task participant $v_i$ in the current vehicle cluster. We consider adding interaction frequency and event timeliness, $R_1' = f_2(R_1, IF_i)$, which represents the prior reputation value of $v_i$ in the local vehicle cluster before the start of this task. As is well known, the higher the interaction frequency between participant vehicles and local vehicle clusters, the more prior knowledge can be provided to calculate their reputation value. Therefore, a higher interaction frequency often reflects the authenticity of the data they provide. The interaction frequency is the ratio of the number of interactions between task participant $v_i$ and local super node RSU to the average number of interactions between task participant and evaluator. The interaction frequency table for task participant *i* is $IF_i$.

4 $$\begin{aligned} IF_i = \frac{N_{i \rightarrow RSU}}{\frac{1}{|M|} \sum _{m \in M} N_{m \rightarrow RSU}} \end{aligned}$$

Where *M* is the number of evaluators in the local vehicle cluster. At the same time, the timeliness of interaction is also considered to affect the credibility of task participant $v_i$, that is, the closer the interaction between $v_i$ and RSU occurs to the current task, the greater the impact on evaluating the credibility of $v_i$, while the interaction over a longer period of time in the past has a smaller impact on it. Therefore, we will use a certain time scale *T* (e.g., one week) as a boundary, and record the number of interactions that occur during time *T* as high trust interactions, denoted as $N_{i \rightarrow RSU}^1$. Interactions that occur beyond time *T* will be recorded as low trust interactions, denoted as $N_{i \rightarrow RSU}^2$. Therefore:

5 $$\begin{aligned} IF_i = \frac{\zeta \cdot N_{i \rightarrow RSU}^1 + \tau \cdot N_{i \rightarrow RSU}^2}{\frac{1}{|M|} \sum _{m \in M} N_{m \rightarrow RSU}}, \quad N_{i \rightarrow RSU} = \zeta \cdot N_{i \rightarrow RSU}^1 + \tau \cdot N_{i \rightarrow RSU}^2, \quad \zeta + \tau = 1 \end{aligned}$$

The trustworthy interaction coefficients *ζ* and *τ* vary with the time scale *T*. Generally, as *T* increases, the high trustworthy interaction coefficient *ζ* decreases and the low trustworthy interaction coefficient *τ* increases. Therefore, the prior credible probability of task participants before applying to participate in collaborative shared tasks is jointly calculated by $R_1$ based on historical task completion status and the interaction frequency $IF_i$ reflecting interaction timeliness:

6 $$\begin{aligned} R_1' = f(R_1, IF_i) = 1 - e^{-\lambda R_1 IF_i} \end{aligned}$$

Among them, $\lambda = -\frac{1}{R_0} \ln \frac{1}{2}$, for vehicles participating in the assistance task for the first time, their prior reputation $R_1' = 0.5$. The $R_1$ value is updated, uploaded, and stored in the latest block after each task is completed, along with the previous task, which can be calculated, making the verification process simpler. At the same time, all interactions and operations are recorded as transactions and stored in the vehicle blockchain. The immutability of the blockchain enables local clusters in a distributed connected vehicle network to update and download interaction records at any time without mutual trust. According to the above formula, the information of each participant in the local vehicle cluster can be calculated.

The $R_1$ value is updated and uploaded together with the *Rep* and *IF* of the previous task after each task is completed, and stored in the latest block. The *Rep* can calculate $R_1$, which makes the verification work simpler. At the same time, all interactions and operations are recorded as transactions and stored in the vehicle blockchain. The immutability of the blockchain enables the RS of each local cluster in a distributed connected vehicle network to update and download interaction records at any time without mutual trust. According to the above formula, the information of each participant in the local vehicle cluster can be calculated.

#### Task matching via MMD and Bayesian inference (R2)

In connected car systems, traditional reputation systems aggregate task matching based on multi-weight subjective logic (TSL) to select collaborative and shared users. The three-weight subjective logic (TWSL) reputation model designed by Jiawen Kang evaluates the matching degree between task participants and tasks from three dimensions: trajectory similarity, interaction frequency, and timeliness. Based on historical performance, $R_1'$ can serve as a good prior knowledge to evaluate the credibility of participants’ data, but it cannot reflect the matching degree between participants and the current task. At the same time, for newly added connected vehicles without historical records, we set their $R_1'$ to 0.5, and it is unfair to only consider historical information for their reputation value. In the traditional federated learning based collaborative sharing model of connected vehicles, the selection of federated learning nodes is mainly based on the matching degree between participants and tasks. However, for distributed and untrusted connected vehicle systems, the data provided cannot be used as trusted knowledge for task matching degree. Evaluating the credibility of participants through historical interaction information in traditional reputation systems as prior knowledge and weighting it with task matching degree will effectively solve this problem. We have designed the second layer reputation value $R_2'$ of a reputation system based on MMD and Bayesian inference, which mainly reflects the degree of matching between the vehicle $v_i$ (local data owned) and the requested task (required data). We obtain the difference in data distribution between participating vehicles and requesting vehicles by differentially weighting the local cluster opinions obtained from aggregating different vehicle clusters.

For the data provider $v_i$’s $R_2'$, obtaining the subjective logic based on the difference in data distribution between the evaluator vehicle $v_j$ and it is used to formulate $v_j$’s personal reputation evaluation of $v_i$. Subjective logic uses the term “opinion” to express the expression of subjective beliefs. The super node RS aggregates the “opinions” of the local vehicle cluster and broadcasts them, with the term $\textit{Local}_i$ being used. The leader selected through a consensus protocol based on Proof of Model Reputation (PoMR) aggregates the “opinions” of all vehicle clusters (with $\textit{Other}_i^k$ in the table) to obtain the task relevance reputation value $R_2'$ for $v_i$.

7 $$\begin{aligned} R_2^i = \rho \cdot \textit{Local}_i + \sum _{k \in K} \rho _k \cdot \textit{Other}_i^k \cdot \frac{1}{\sum _{k \in K} \rho _k} \end{aligned}$$

In the Internet of Vehicles, the high mobility of vehicles results in a significant correlation between the data correlation evaluation and distance of collaborative shared task vehicles in different vehicle clusters. $\rho _k$ reflects the geographical location difference between the vehicle cluster of $v_i$ and the vehicle cluster *K*, where $\rho _k = b + e^{-d_k}$. Here, *b* is a distance adjustment bias term, and $d_k$ represents the relative distance between vehicle cluster *K* and $v_i$. $d_k = \frac{1}{M_k} \sum d_{k_j}$, where $M_k$ and $d_{k_j}$ respectively represent the number of evaluators in the vehicle cluster and the distance between $v_j$ and $v_i$. Vehicle-cluster MMD. We measure the matching degree between the participants and the current task by using the differences in data distribution among them, and then obtain evaluations of data quality and learning quality, known as “opinions”. Compared to traditional subjective logic with a single weight, we consider formulating local opinions based on different weights. $\mathcal {F} = \{f_1, f_2, \dots , f_n\}$ is a metric function for the difference in data distribution required for different collaborative training tasks in the Internet of Vehicles.

Assuming that the evaluator vehicle $v_j$ evaluates the similarity between the data provided by vehicle $v_i$ and the task request data, $v_i$ performs a data distribution hypothesis test on the local data, broadcasts the obtained local data distribution $g_i = \{x_1, x_2, \cdots , x_m\}$ to the evaluator vehicle $v_j$, and then $v_j$ uses its own local data distribution $g_j = \{y_1, y_2, \cdots , y_n\}$ to provide local opinions on the $v_i$ data.

8 $$\begin{aligned} \mathcal {MMD}^2_{i \rightarrow j}[\mathcal {F}, X, Y] = \sup _{f \in \mathcal {F}} \mathcal{M}\mathcal{D}^2(g) = \sup _{f \in \mathcal {F}} \left( \frac{1}{m} \sum _{i=1}^{m} f(x_i) - \frac{1}{n} \sum _{j=1}^{n} f(y_j)\right) ^2 \end{aligned}$$

Measure the data similarity of participants by calculating the maximum mean square difference in data distribution between both parties. The performance of the distribution difference test function varies for different task data. Compared to the mean, variance, and other differences of a single comparison data distribution, after aligning the sample size, the function set $f_i \in \mathcal {F}$ is searched for a value that maximizes $\mathcal{M}\mathcal{D}^2(g)$ to better reflect the data differences between the participating parties. Therefore, after normalization, the following equation represents the local opinion of the evaluator vehicle $v_j$ on the task data provided by vehicle $v_i$.

9 $$\begin{aligned} \delta _{i \rightarrow j} = \lambda _2 \cdot e^{-r \cdot \mathcal {MMD}^2_{i \rightarrow j}[\mathcal {F}, X, Y]} \end{aligned}$$

where $\lambda _2$ is the scaling factor used to map the MMD value to the confidence of the local opinion.

After calculating the weights of all evaluators’ opinions, they are combined into a common opinion of the local vehicle cluster through Bayesian inference. Under the local opinion evaluation of vehicle cluster *M*, the comprehensive matching degree between the data provided by vehicle $v_i$ and the data required for the collaborative sharing task published by vehicle $v_j$ is:

Local$$_i = P(\text {opinion}_{i \rightarrow j} \mid \text {opinion}_{m \rightarrow i})$$.

10 $$\begin{aligned} \begin{aligned} A&= P(\text {opinion}_{i \rightarrow j}) \cdot \prod _{m \in M} P(\text {opinion}_{m \rightarrow i} \mid \text {opinion}_{i \rightarrow j}), \\ B&= P(\overline{\text {opinion}_{i \rightarrow j}}) \cdot \prod _{m \in M} P(\text {opinion}_{m \rightarrow i} \mid \overline{\text {opinion}_{i \rightarrow j}}), \\ P(\text {opinion}_{i \rightarrow j} \mid \text {opinion}_{m \rightarrow i})&= \frac{A}{A + B}. \end{aligned} \end{aligned}$$

Among them, $P(\text {opinion}_{i \rightarrow j}) = \delta _{i \rightarrow j}$, where all evaluator vehicles $v_m$, *m ∈ M* belong to a vehicle cluster and are independent of each other. By calculating the maximum difference in the data distribution provided by vehicle $v_m$, the evaluator’s local opinion on the data distribution provided by vehicle $v_i$ is obtained. The credibility $R_1^i$ of vehicle *i* before the start of this collaborative sharing task is the prior probability, and after weighting, the evaluator’s vehicle $v_j$’s comprehensive opinion on vehicle $v_i$’s participation in this collaborative sharing task is obtained, namely:

11 $$\begin{aligned} P(\text {opinion}_{m \rightarrow i} \mid \text {opinion}_{i \rightarrow j}) = \delta _{m \rightarrow i} \cdot R_1^i \end{aligned}$$

12 $$\begin{aligned} P(\text {opinion}_{m \rightarrow i} \mid \overline{\text {opinion}_{i \rightarrow j}}) = 1 - \delta _{m \rightarrow i} \cdot R_1^i \end{aligned}$$

#### Multi-layer reputation algorithm

Before the vehicle starts the collaborative sharing task, the reputation value is initialized by executing smart contracts on the vehicle blockchain. The *Type = 1* table requests data sharing tasks, and the vehicle shares data with the management node. Because the management node can verify the quality of the vehicle’s shared data locally, we evaluate the reliability of the data provided by the vehicle based on its historical performance reputation value $R_1$. This ensures the authenticity and reliability of the shared data in a distributed system that cannot be fully trusted, while reducing communication and evaluation consumption. For *Type = 2*, the request is for collaborative sharing tasks. We will use the reputation value $R_1$ based on the vehicle’s historical performance as the prior credibility, and consider the reputation value $R_2$ of multi-weight subjective logic to evaluate the vehicle’s collaborative ability. For different collaborative requests of different tasks, the higher the matching degree of participating vehicles, the better the quality of collaborative sharing results. Therefore, $R_2$ as the retrieval weight for vehicle *i* participating in collaborative sharing tasks can greatly improve the training quality of the entire distributed system.

**Algorithm 1:**
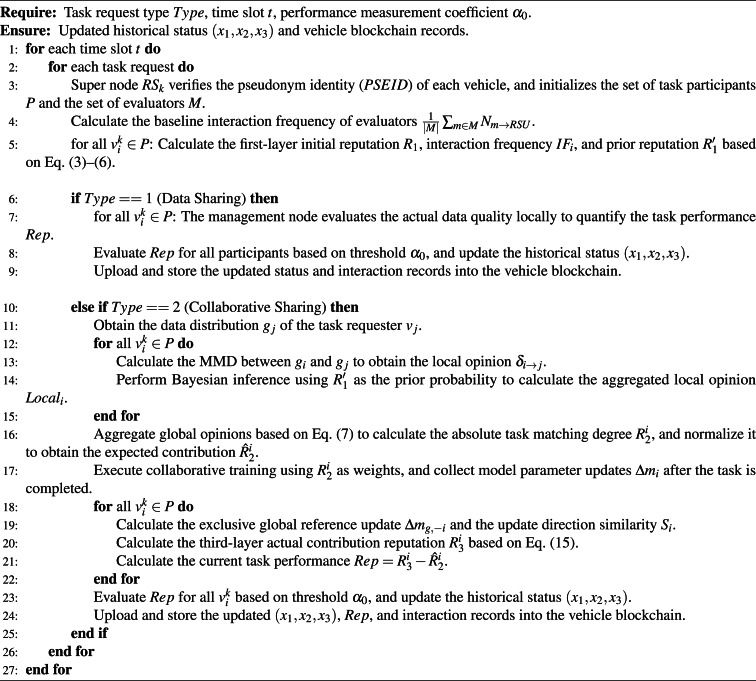
Multi-layer reputation evaluation and update.

#### Contribution evaluation ($R_3$)

In the blockchain assisted vehicle networking, the contribution evaluation of collaborative sharing quality is uploaded to the blockchain after distributed consensus and used for data pricing and reputation updates. In our three-layer reputation system, the reputation of vehicles is measured by their historical performance, multi-weight subjective evaluation, and latest task performance, and the training quality evaluation of this task is the key to updating the vehicle reputation value. The contribution evaluation results of vehicles in the vehicle cluster will be verified by the local super node RS and uploaded to the blockchain after consensus. The contribution of vehicles participating in collaborative sharing tasks is often evaluated.

Traditional loss-based contribution evaluation (such as MAE) tends to suffer from numerical inflation under noise, thereby falsely penalizing honest nodes. To address this, we introduce GNN to extract node connection weights as “feature fingerprints” and evaluate contribution by assessing the directional consistency between local model updates and aggregated global updates.

Here, the update vector of model parameters reflects the changes in the aforementioned GNN connection weights. Let $\Delta m_i$ denote the parameter update of the local model trained by vehicle $v_i$. The global reference update $\Delta m_g$ used to evaluate vehicle $v_i$’s contribution leverages the preceding second-layer task matching reputation $R_2$ as a weight, and aggregates updates from other vehicles after excluding vehicle $v_i$ itself:

13 $$\begin{aligned} \Delta m_g = \sum _{k=1, k\ne i}^{N} \frac{R_2^k}{\sum _{j=1, j\ne i}^{N} R_2^j} \Delta m_k \end{aligned}$$

where $R_2^k$ denotes the reputation value obtained by vehicle *k* in the second layer. We define the update direction similarity of the vehicle as:

14 $$\begin{aligned} S_i = \frac{\Delta m_i^T \Delta m_g}{(\Vert \Delta m_i\Vert _2 + \epsilon )(\Vert \Delta m_g\Vert _2 + \epsilon )} \end{aligned}$$

where *ε> 0* is a small constant introduced to avoid division by zero. A larger similarity score $S_i$ indicates that vehicle $v_i$’s local update is more aligned with the aggregated reference direction formed by high $R_2$ nodes, and thus its contribution to collaborative training is more reliable.

To jointly reflect update alignment and data volume support, vehicle $v_i$’s third-layer reputation value is defined as:

15 $$\begin{aligned} R_3^i = \frac{|d_i|^\alpha \exp (\lambda _3 S_i)}{\sum _{j=1}^{N} |d_j|^\alpha \exp (\lambda _3 S_j)} \end{aligned}$$

where $|d_i|$ is the volume of training data owned by vehicle $v_i$, $\alpha \in [0, 1]$ controls the impact of data volume on contribution evaluation, $\lambda _3> 0$ is a temperature parameter used to adjust the sensitivity to directional similarity, and *N* is the number of vehicles participating in the current collaborative sharing task. This formula satisfies $R_3^i \in (0,1)$ and $\sum _{i=1}^N R_3^i = 1$, thus $R_3^i$ can be interpreted as a normalized contribution weight.

The third-layer reputation value $R_3^i$ of vehicle $v_i$ is a weight used to evaluate the contribution of its training model. In our three-layer reputation system, we consider the difference between the contribution degree $R_3$ of the vehicle after completing the collaborative sharing task and the task matching degree $R_2$ before training. After the vehicle completes the training task, the super node RS of the local vehicle cluster updates the new reputation value and uploads it to the blockchain, maintaining the records in the reputation table *Rep*. In subsequent collaborative contribution tasks, the new $R_1$ can be calculated based on *Rep*.

### Hybrid vehicle blockchain and DAG scheme

#### Federated learning model based on the hybrid scheme

In our hybrid federated learning scheme, data is trained locally without sharing, transforming the collaborative sharing problem in the Internet of Vehicles into model sharing. This not only protects user data privacy and security, but also integrates all participating vehicle training models in the vehicle cluster as global model iteration parameters, greatly reducing the communication consumption of global synchronous iteration.

The vehicle blockchain runs on all management and regulatory committee nodes, while the local DAG runs on the vehicle nodes. The vehicle blockchain records all interaction events between vehicles, between vehicles and RSU, and between RSU and cloud service providers. At the same time, vehicles register with certificate issuing agencies (RSU or Cloud) to obtain certificates for participating in V2X data sharing. In order to achieve efficient and trustworthy collaborative sharing tasks, our proposed hybrid consensus protocol is divided into local model iterative consensus (DAG consensus) for vehicle clusters and distributed consensus (PoMR) for vehicle blockchain. The management committee node (RSU) and regulatory committee node (Cloud) in the vehicle blockchain are responsible for managing the synchronous federated learning global model aggregation process, verifying the generated interactive information, and serving as the consensus and verification nodes of the vehicle blockchain to achieve block publication for the entire vehicle network. After the CAV in each vehicle cluster verifies and updates the local DAG, the V2V broadcasts the local DAG to achieve asynchronous iterative updates of the local model $Model_k$. When the model meets a certain running time or iteration round, the management node RSU of the vehicle cluster is responsible for verifying the local DAG and aggregating the local federated learning model. The management node (RSU) supervises the DAG network of the local vehicle cluster to ensure the aggregation and updating of collaborative training on the vehicle side.

In the Internet of Vehicles, the high mobility of vehicles also makes it difficult to achieve stable data transmission. At the same time, due to the immutability of vehicle blockchain, it is necessary to prioritize the authenticity and validity of new block data, which cannot meet the requirements of efficient model iteration in federated learning. In our hybrid approach, the collaborative sharing task $Req_j$ requested by the vehicle requester is transformed into an asynchronous federated learning task, which means that the collaborative sharing task output is a global federated learning model $Model_t = f(Req_j)$ jointly completed by multiple participating vehicle clusters $VCluster_p \subset VC$ that meets the accuracy requirements.

The federated learning process of our hybrid approach divides model aggregation into local aggregation executed on the vehicle side and global aggregation executed on the cloud hosting node. The time consumption of synchronous federated learning model iteration is determined by the node with the maximum sum of computation consumption $T_{cp}$ and communication consumption $T_{cm}$. The dispersion of vehicles in the Internet of Vehicles has a negligible computational cost compared to communication cost. However, the communication efficiency between RSU and the cloud is relatively stable and efficient. Therefore, in order to reduce traffic consumption and improve aggregation efficiency, we only perform synchronous aggregation between RSU and the cloud, and the iteration consumption is determined by the minimum training communication consumption of the vehicle cluster:

16 $$\begin{aligned} \max _{i \in V_p}(T_{cp}(v_i) + T_{cm}(v_i)) \ge \min _{k \in K} \left\{ \max _{v_i^k \in V_k}(T_{cp}(v_i^k) + T_{cm}(v_i^k))\right\} \end{aligned}$$

Each vehicle cluster updates the local DAG to iterate the local model, achieving multiple model updates and iterations between two communication rounds between RSU and the cloud. In our training process, we iteratively improve the accuracy of the model by minimizing the global loss function *F*(*w*) in the direction of gradient descent. At the same time, differential privacy is utilized to add perturbations and disturbances to the parameter *w* of the global model of the interaction between the management node and the cloud chairman node, which will avoid the leakage of raw data caused by inference attacks. Following Wei et al.^13^, in the empirical evaluation we instantiate this protection under the $(\epsilon , \delta )$-DP setting, where $\epsilon \in \{50, 60, 80, 100\}$ and *δ = 0.01*. The variance of the injected local noise is calibrated according to the trainable parameter dimension *d*, which scales linearly with *d*. Therefore, due to the parameter-efficient design of our architecture, the required noise magnitude under the same privacy budget is correspondingly reduced.

17 $$\begin{aligned} \begin{aligned}&\min _{w \in R^d} F(w) = \frac{1}{|V_k|} \sum _{k \in K} C_k \cdot F_k(w) \\&s.t. \quad Pr(w_i \in R^d) \le \exp (\varepsilon ) Pr(w_i' \in R^d) \end{aligned} \end{aligned}$$

To improve the overall training efficiency of distributed systems, the time cost of participating in collaborative shared vehicles should meet the following constraints. In the node selection process, we mainly consider vehicles with low time cost to improve the speed of overall synchronization iteration.

18 $$\begin{aligned} \sum _{i=1}^{t} \Delta t(i) \le \min _{k \in K} \left\{ \max _{v_i^k \in V_k}(T_{cp}(v_i^k) + T_{cm}(v_i^k))\right\} \end{aligned}$$

The above *t* is the maximum local iteration number of the vehicle cluster, and *t*(*i*) is the time required to execute the *i*-th iteration, which ensures that all vehicle clusters can synchronously iterate the global model and connect to RSU during the learning communication phase.

Our hybrid federated learning approach aims to train a federated learning model based on distributed datasets. The specific process of the entire federated learning task is as follows:

Node selection and initialization: Firstly, the chairman of the regulatory committee receives reputation update information from various vehicle cluster management nodes regarding the vehicles applying to participate in the task. At the same time, a global machine learning model and initialization parameter $u_{ini}$ are selected to initialize the federated learning process. To improve collaboration efficiency and quality, node selection is based on the communication capabilities of different vehicle clusters to select nodes with higher computing resources to participate in the training task. Specifically, the chairman of the regulatory committee executes a reputation partition node selection algorithm based on DRL to select vehicles (trainers) *VP ⊂ VC* with high computing and communication capabilities $C_{p_i} \cdot C_{m_i}$ that meet different reputation thresholds $R_2$ to participate in model training. Then, based on the vehicle cluster aggregation score *W*(*RS*) in Eq. (23), the parameter vector $W_U$ is allocated to each containing training vehicles according to the aggregation score *W*(*RS*). The vehicle cluster management node $RS_k$.

Local training: During the model training process, the data owned by each participant will not leave the data owner. At the same time, when the distributed gradient descent method is used to update the DAG at the vehicle end to achieve local model iteration, the information related to the federated learning model is transmitted and exchanged between all parties in an encrypted manner. This ensures that no participant can infer the original data of other parties, further guaranteeing the privacy and ownership of the data of all parties. Each vehicle cluster participates in vehicles $v_i^k \in V_k$ uses local data $D_i^k$ to train a local model $u_i^k(t)$ based on $t_k$, specifically by retrieving local models of other participating vehicles from the local DAG for each vehicle, and calculating local gradient descent $\nabla F_i(u_t^k)$ to iterate the local model. Then, the vehicle $v_i^k$ adds its local model $u_i^k(t)$ as a transaction that needs to be validated to the local DAG. This process is repeated by all participating vehicles in the local vehicle cluster until the global model of the local vehicle cluster meets the iteration requirements. Each vehicle cluster participates in vehicles $v_i^k$ uses local data $D_i^k$ to train a local model $w_i^k(t)$ based on $t_k$, specifically by retrieving local models of other participating vehicles from the local DAG for each vehicle, and calculating local gradient descent $\nabla F_i(w_t^k)$ to iterate the local model. Then, the vehicle $v_i^k$ adds its local model $w_i^k(t)$ as a transaction that needs to be validated to the local DAG. This process is repeated by all participating vehicles in the local vehicle cluster until the global model of the local vehicle cluster meets the iteration requirements.

19 $$\begin{aligned} w_i^k(t) = w^k(t) - \alpha \nabla F_i(w_{t-1}^k) \end{aligned}$$

Global aggregation: The management node RS of each vehicle cluster serves as a local server to verify and aggregate the model training results $w^k(T)$ of the participating vehicles $v_i^k \in V_k$ in the local cluster. The chairman node in the cloud serves as a global model server to retrieve the updated model parameters $w^k(T)$ of each vehicle cluster from the vehicle blockchain, and performs global aggregation through weighting to achieve the update *w(T+1)* of the global model. The model iteration of each vehicle cluster aggregates the global model as follows:

20 $$\begin{aligned} w(T + 1) = \frac{\sum _{k=1}^{N} C_k w^k(T)}{\sum _{k=1}^{N} C_k} \end{aligned}$$

The $C_k$ table shows the contribution of each vehicle cluster to the global model in the *t*-th iteration, and the aggregation weight is obtained based on the third layer reputation value $R_3$ of the participating vehicles in each vehicle cluster.

#### PoMR-based consensus in vehicle blockchain

Based on the Proof of Model Reputation (PoMR) consensus protocol, we integrate the reputation mechanism into federated learning, block based on reputation values, select leading nodes, and combine the reputation mechanism with node selection in federated learning to evaluate federated learning nodes based on reputation values.

The decentralized data sharing solution combined with blockchain not only guarantees the data ownership of distributed nodes, but also has high system operational redundancy. But how to improve consensus efficiency and reduce consensus costs is the key to running blockchain in data sharing of the Internet of Vehicles. Traditional PoW and PoS both require solving mathematical problems, which can result in serious waste of computing resources. At the same time, their consensus efficiency cannot meet the requirements of asynchronous data sharing in the Internet of Vehicles. Traditional DPoS, on the other hand, represents nodes to vote and select committees based on the maintained blockchain tokens. Although it improves consensus efficiency, it faces the risk of centralization due to the human factors of token holders. To address the above difficulties, we propose a vehicle blockchain consensus algorithm based on DPoS combined with a three-layer reputation system, which combines voter reputation values - a consensus protocol based on Proof of Model Reputation (PoMR).

The PoMR consensus protocol combines the collaborative sharing task matching process with the consensus process of the vehicle blockchain. Vehicles do not participate in the consensus of block generation, but instead delegate the management node RSU of each vehicle cluster to serve as the consensus representative. The RSU, who becomes the consensus representative, manages the operation of the vehicle blockchain by setting the management block interval and block size. At the same time, consensus representatives also play a role in verifying and aggregating local models in local DAG transactions. The consensus of vehicle blockchain is achieved by the management nodes of each vehicle cluster representing the participating vehicles, reducing communication overhead by sending consistent messages only to the management committee nodes and regulatory committee nodes instead of all nodes. However, the reduction in the number of nodes also poses a challenge to the reliability of consensus reached. Therefore, to increase system security, in each block verification slot, the Leader is selected based on the task performance of each RSU and reputation voting from participating vehicles in the local cluster. Then, the remaining consensus nodes and regulatory committee nodes act as block validators to verify transactions in the block and return the audit results to the leadership. The leader collects all received audit results to determine whether to submit candidate blocks. If the candidate block passes the verification of all regulatory committee nodes and more than half of the consensus nodes, the leader will broadcast the new block for updating and storing in the vehicle blockchain.

Step 1: Selection of Leaders in Consensus Nodes. The consensus selection of leaders in the consensus nodes depends on the quality of the global federated learning model and the reputation voting of vehicles in the local vehicle cluster. Specifically, each management committee node verifies the training quality of the locally aggregated global model based on the test set during collaboration. We use prediction accuracy to quantify the performance of the trained local models of the vehicle cluster, and weight the competitive leaders based on the aggregated reputation $Rep_{RS_k}$ of each vehicle cluster. The reputation value of the vehicle cluster is aggregated from the second layer reputation value $R_2$ of the participating vehicles updated by the regulatory committee. The Leader node in the consensus packages all data interaction records into a new block. Specifically, the management node $RS_k$ verifies the requests submitted by local vehicles to participate in collaborative training transactions and publishes tasks. At the same time, it obtains the historical reputation value $R_1$ of the vehicle and the aggregated opinion *k* of the local vehicle cluster *k* based on the reputation algorithm (Algorithm 1), and packages it together with the request to participate in collaborative training transactions, accompanied by the corresponding certificate ($Cert_{RS_k}$) registered by the authoritative institution, the private key $SK_{RS_k}$ signature, and timestamp. Then, it encrypts and sends it to the supervisory committee chairman node $Cloud_l$. It obtains the updated vehicle reputation list $R \rightarrow R_2$ from the supervisory committee chairman node $Cloud_l$. The score $W(RS_k)$ of the management node $RS_k$ is generated based on the resources and performance of the participating vehicles $R_2$ in the vehicle cluster and the management node $RS_k$, and selects the management node as each round according to its size, the leader in consensus.

21 $$\begin{aligned} RS_k \rightarrow CM_t : Request = E_{PK_{CM_t}}(Transaction||R_1'||opinion_k||Cert_{RS_k}||Sig_{SK_{RS_k}}||timestamp) \end{aligned}$$

The chairman node of the regulatory committee updates the reputation information $R \rightarrow R_2$ of participating vehicles in the cloud and provides feedback to all management nodes.

22 $$\begin{aligned} CM_t \rightarrow All : Reply = (Test||Reputation_{list}||Cert_{CM_t}||Sig_{SK_{CM_t}}||timestamp) \end{aligned}$$

Considering the impact of different vehicle reputation values on the aggregated reputation of vehicle clusters, we can divide the second layer reputation value $R_2$ based on task matching degree into three layers: highly matched, matched, and negatively correlated. We use a segmentation function $s_i$ for vehicle reputation, where *ρ* is a reliable boundary that can be adjusted according to the task. In this round of collaborative training, the reputation value of the vehicle cluster management node is the segmented weighted second layer reputation value of its participating vehicles $i \in V_k$. We consider the task performance $F_{RS_k}$ of the vehicle cluster management node $RS_k$ to be related to its communication, computing, and storage capabilities certified by authoritative institutions.

23 $$\begin{aligned} W(RS_k) = F_{RS_k} \cdot \frac{\sum _{i=1}^{N_k} R'_{i2} \cdot s_i}{N_k}, \quad s_i = {\left\{ \begin{array}{ll} (1-q)p, & \text {if } R'_{i2} \ge \rho \\ (1-q)(1-p), & \text {if } 0.5 \le R'_{i2}< \rho \\ q, & \text {if } R'_{i2} < 0.5 \end{array}\right. } \end{aligned}$$

The aggregation score $W(RS_k)$ of each vehicle cluster RS is arranged from high to low, and the consensus node with higher score is selected according to the consensus cycle to become the leader to promote the consensus process of the vehicle blockchain.

Step 2: Generation and Contribution Evaluation of Transaction Records. In each collaborative task, all interaction records are verified and signed in the form of transactions before being sent to the Leader. The collaborative sharing contribution $R_3$ of vehicles is evaluated by the management node and broadcasted to all regulatory committee nodes in the form of reputation contribution transactions. The regulatory committee nodes act as validators of the contribution evaluation and calculate $MAE(m_i)$ and $R_3^i$ based on the $m_i$ of each participating vehicle training model in the transaction. If the calculated $R_3$ is within a certain range, approval will be sent to the chairman node. If a shared reputation $R_3^i$ is approved by all regulatory committee nodes, the chairman node will send the corresponding token to participating vehicle *i*.

Step 3: Generation and verification of new blocks. The leader verifies and packages the collected transaction records into new blocks, which are broadcasted to the remaining management committee nodes and all regulatory committee nodes as block validators for review and approval (such as block format, hash, and timestamp). The Leader receives all audit results, and if all regulatory committee nodes and more than half of the management nodes serving as block validators approve the review, the Leader sends the signed new block data to all nodes for synchronization. Finally, these data will be stored in the vehicle blockchain.

#### Local DAG for quality verification in vehicle clusters

We utilize local DAGs in vehicle clusters to improve the efficiency of federated learning in latency-sensitive collaborative sharing tasks in the Internet of Vehicles. DAG is saved locally by vehicles, continuously propagated by vehicles through gossip, and updated asynchronously to achieve asynchronous consistency with other vehicle nodes. Asynchronous consistency allows vehicles to reach agreements on historical states rather than current states. In the vehicle cluster, local training model parameters, vehicle validation contribution weights, and interaction information between vehicles are recorded as transactions and stored as DAG nodes. The connection between node *i* and node *j* established through the approval relationship between transactions is denoted as DAG edge: $n_i \rightarrow n_j$. The relevant transactions shared in the local DAG of each vehicle are periodically synchronized to the management node of the vehicle cluster, verified and aggregated, and uploaded to the vehicle blockchain.

**Fig. 4:**
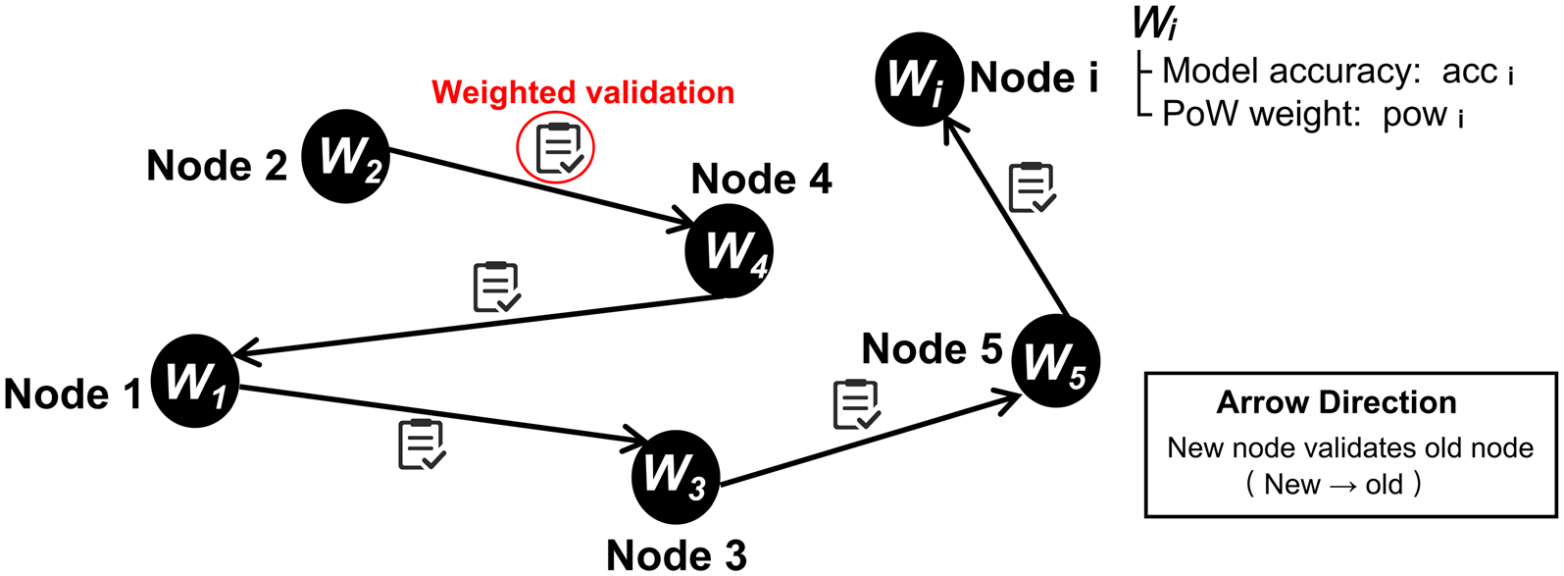
DAG construction in a local vehicle cluster.

To improve the quality of collaboration and sharing in vehicle clusters, the approval between DAG nodes relies on vehicle model updates to establish quality evaluation and verification weights, as shown in Fig. 4. At the same time, we reduce the additional cost of quality verification through reputation partitioning. The update of federated learning local models in vehicle clusters relies on the confirmation of the “heaviest branch” in the DAG. The basis of consensus is that most vehicles have honest results. Distributed verification computation in the DAG is equivalent to simplified PoW, and vehicles obtain the right to add tasks by calculating the simplified PoW. The participating vehicle $v_i$ transmits its local training model $m_i(t)$ parameters to nearby vehicles in a transactional form through V2V communication for local updates. The vehicles add new transaction nodes to the local DAG based on reputation block quality evaluation.

The management node of the vehicle cluster obtains the reputation linked list (*Rlist*) of all participating vehicles from the chairman node of the regulatory committee. According to task requirements, it partitions the second-layer reputation values of vehicles from high to low, where $Rlist=\{(i,\textrm{Rep}_i)\}$ and $\textrm{Rep}=\{block_1,block_2,\ldots ,block_l\}$. Vehicle *i* adds new transaction nodes to the local DAG, including a local model $m_i(t)$ trained on local data and two approved transaction references selected on the local DAG through the validation process. The cumulative weight of each DAG node is determined by the reputation of the publishing vehicle and the contribution of transaction verification.

Since model iteration among vehicles can be regarded as a Markov process, the Markov Chain Monte Carlo (MCMC) method is used to simulate the transaction selection process at each step. Vehicles use weighted transition probabilities to select transactions with high cumulative weights for verification. Specifically, the weighted random walk follows the “high cumulative weight” strategy, in which the next step is biased toward transactions with higher cumulative weights. Through weight-biased random walks, vehicles select nodes with a high probability of becoming confirmed transactions for verification. The simulation of MCMC is based on:

24 $$\begin{aligned} E[f(x)] \approx \frac{1}{m} \sum _{i=1}^{m} f(x_i), \quad (x_0, x_1, \cdots , x_m) \sim MC(p) \end{aligned}$$

Among them, *MC*(*p*) is a Markov chain generated by a Markov process. The confirmation of DAG transaction nodes is based on their cumulative weights. When a new transaction is published, two walkers are placed on the DAG to select two transactions with higher cumulative weights for validation. A walker moves from the current transaction node *x* to a candidate neighboring transaction node *y* with transition probability $P_{xy}$:

25 $$\begin{aligned} P_{xy} = \frac{e^{W(y)}}{\sum _{z \in N(x)} e^{W(z)}} \end{aligned}$$

where *y ∈ N(x)*, *N*(*x*) denotes the set of candidate neighboring transactions of node *x*, and *W*(*y*) denotes the cumulative weight of transaction node *y*.

The DAG is broadcast by vehicles through gossip, and nearby vehicles $v_i$ select the vehicle set in the highest-reputation block to perform MCMC-based transaction selection and verification. The cumulative weight of each DAG transaction node is determined by the training model accuracy of its publisher vehicle and the verification contribution, denoted as $W(m_i(t))$:

26 $$\begin{aligned} W(m_i(t)) = \frac{|d_i| + \sum _j d_{m_j}}{\sum _{k=1}^{N_k} |d_j| + \sum _j d_{m_j}} \cdot Acc_i(m_i(t)) + \frac{1}{n} \sum _{j=1}^{n} \frac{R_j}{2} \cdot W(j) \end{aligned}$$

where $d_i$ is the data size of vehicle *i*, $d_{m_j}$ is the cumulative data size of the local aggregation model $m_j(t)$, and *W*(*j*) is the verification contribution of transaction *j*. DAG consensus refers to the establishment of the “heaviest branch”. Honest nodes preferentially choose transactions with higher cumulative weights for verification to increase the weight of their added transactions, while branches with lower weights will eventually tend to be isolated. By using reputation partitioning, we simplify the DAG consensus process, as higher second-layer reputation implies higher task-matching quality, which improves collaborative training efficiency. The vehicle obtains the right to add transactions through local DAG transaction verification, which provides the DAG with protection against malicious attacks such as spam attacks. On the other hand, the simplified PoW has much lower complexity than traditional PoW, with lower computational resource costs and higher efficiency. As the number of transactions increases, more verifications can be performed, which can further improve the efficiency and security of the DAG.

### DRL-based participant selection

For a collaborative sharing request, we execute a deep reinforcement learning based node selection algorithm in the management node $RS_k$ of each vehicle cluster, that is, select a subset $v_p^k \subset V_k$ of participating vehicle nodes in each vehicle cluster to participate in federated learning based collaborative sharing. The goal is to improve the quality of federated learning models in the distributed system of the Internet of Vehicles, which is not trusted by each other.

The efficiency of the global aggregation stage in federated learning models depends on the computational and communication efficiency of all nodes. Therefore, in traditional federated learning node selection algorithms for connected vehicles, vehicle nodes with high communication efficiency and strong computing power are considered to reduce aggregation time. Lu et al.^36^ consider minimizing execution time and maximizing aggregation accuracy. However, in the distributed system of the Internet of Vehicles, which lacks mutual trust, it is necessary to consider the selection weight of vehicles’ participation in federated learning contribution evaluation. Secondly, node selection algorithms are executed before distributed training, and vehicles evaluate the aggregation accuracy based on the accuracy of the model training. The shared method cannot improve the sharing efficiency and quality of a complementary trust network. Therefore, we introduce our reputation system into the node selection algorithm of Lu et al.^37^. Vehicles are segmented based on the second layer reputation value $R_2$, and the threshold $R_{tv}$ for selecting vehicle reputation values for participating nodes is determined by the differences in timeliness and training costs for different task requests *Req*. The threshold $Req: R_{to}$ for selecting vehicle reputation values for participating nodes is determined by the differences in timeliness and training costs for different task requests *Req*. A higher reputation threshold $R_{tv}$ is used for node selection. Although the vehicle set is smaller, the correlation and credibility between vehicles and tasks are higher, and vice versa.

We express the node selection algorithm for vehicles as a combinatorial optimization problem, selecting high computational and low communication vehicles from the vehicle set $V_{R_{tv}}=\{i \in V, R_{i2} \ge R_{tv}\}$ that satisfy the reputation threshold, in order to minimize the total cost in federated learning, i.e., the global iteration time cost *T* input. At time *t*, the vehicle selection status in the vehicle set $V_{R_{tv}}$ is listed as input $t=(input_{t1}, input_{t2}, \cdots , input_{tn})$, $input_{ti} \in \{0, 1\}$, respectively, indicating that vehicle $v_i$ has not been selected and has been selected.

We use DRL based machine learning algorithms to find the optimal set of participating vehicles to assist in training. The global iteration time is determined by the vehicle cluster that spends the most time in each iteration. The calculation cost of the vehicle, $T_{cp}$, and the communication cost, $T_{cm}$, are taken as time costs. Among them, $C_{p_i}$ and $C_{m_i}$ are the available computing resources (CPU cycles/s) and communication transmission rate, respectively. $|d_i|$ and $|w_i|$ are the local data size and transmission model parameter size used for the current training model. The global time cost is determined by the vehicle cluster with the highest time cost. Firstly, we define the time cost of vehicle cluster *k* in node selection algorithm *t* as:

27 $$\begin{aligned} T^k(\lambda ^t) = \max _{v_i^k \in V_{R_{tv}}} \left( \frac{|d_i| \cdot \alpha _m}{C_{p_i}} + \frac{|w_i|}{C_{m_i}}\right) \end{aligned}$$

Therefore, we can define the time expenditure function as:

28 $$\begin{aligned} T(\lambda ^t) = \frac{1}{|RS|} \sum _{k=1}^{|RS|} T^k(\lambda ^t) \end{aligned}$$

Among them, |*RS*| is the number of all vehicle clusters. $IR_{sl}$ is the number of all vehicle clusters. The combinatorial optimization problem of node selection transformation can be expressed as:

29 $$\begin{aligned} \min _{\lambda ^t} T(\lambda ^t), \quad s.t. \quad \lambda _i^t \in \{0, 1\}, \forall i \end{aligned}$$

We use DRL to solve the node selection problem mentioned above. DRL learns models through interaction with the environment and does not require prior training data or model assumptions. We utilized Deep Deterministic Policy Gradient (DDPG) to find the optimal solution for node selection in asynchronous federated learning.

### Secure collaboration and sharing process

Our vehicle road cloud chain system provides a more secure and feasible solution for distributed vehicles to achieve collaborative sharing in the Internet of Vehicles. The vehicle blockchain ensures tamper proof and traceable interactions, while relying on transparent and open consensus mechanisms to avoid data security risks brought by traditional centralized data storage. In the context of data sharing, in order to avoid privacy and security risks associated with data holders sharing raw data, federated learning is combined to only share collaborative results, protecting the privacy of data holders. However, in a vehicle environment where the Internet of Vehicles is not trusted with each other, the quality of shared collaboration results depends on the honesty of the shared vehicles, which is clearly unreliable. We quantify the honesty attribute of vehicles through a three-layer reputation evaluation, and combine it with the collaborative sharing process and vehicle blockchain consensus process. This is beneficial for improving the quality of the entire collaborative sharing task results and incentivizing vehicles with high-quality collaborative capabilities to actively contribute, avoiding free riding behavior of some vehicles.

In our vehicle road cloud chain system, the specific steps for secure collaboration and task sharing in each time slot are as follows:

1. Identity registration and authentication stage. To achieve a balance between protecting users’ privacy information and ensuring shared security in the Internet of Vehicles, vehicle users submit their identity information to trusted authorities (such as transportation departments) and obtain their unique identity $ID_i$ after authentication. Due to the transparent nature of vehicle blockchain, different users have different privacy protection requirements. In order to reduce the correlation between collaborative sharing tasks completed by the same vehicle and avoid identity attacks by malicious vehicles’ inference, in the vehicle blockchain, vehicle $v_i$ can generate different pseudonyms $PSEID_i$ based on its unique identity $ID_i$, and obtain corresponding public key $PK_{PSE_i}$ and private key $SK_{PSE_i}$. Authoritative institutions verify the identity $ID_i$ sent by vehicle *i* and the corresponding public key $PK_{PSE_i}$, and then send the authenticated certificate $Cert_{PSE_i}$. All vehicles with certification certificates can participate in collaborative sharing in the vehicle road cloud chain system. The randomly generated chairman node Cloud of the regulatory committee within the time slot $T_c$ is authenticated by authoritative institutions and broadcasts identity authentication certificates to all regulatory and management committee nodes. It is responsible for coordinating and updating the global vehicle reputation table and iterating the federated global model in collaborative sharing tasks within the time slot, while executing node selection algorithms to select participating nodes.
2. Task request update and task initialization phase. The task requester *j* sends a task request *Req* to the management node $R_k$ of the local vehicle cluster in the form of a transaction (Table 1). The management node $R_k$ verifies the transaction request (verifies the authentication certificate of the authority). At the same time, $RS_k$, as the consensus node of the vehicle blockchain, first checks whether the task *Req* exists in the vehicle blockchain. If so, it initiates a result sharing request to the previous management node that sent the task. The request passes and the task result is returned to the requester. Otherwise, the management node $RS_k$ broadcasts the task request in the local vehicle cluster and shares it with all consensus nodes. The received vehicles send a request to participate in collaborative sharing tasks (Table 2) to the local management node to complete task initialization.
3. Multi-party reputation evaluation stage. The management node of the vehicle cluster obtains the historical reputation value *Rep* of local participating vehicles from the vehicle blockchain, and completes the calculation of the reputation value $R_1$ based on historical information of the vehicles through the interactive evaluation of local vehicles. Then the management committee node $RS_k$, *k=1,2,…*, completes the update of the reputation list of vehicles applying to participate in this collaborative sharing task ($R \rightarrow R_2$) by interacting with the supervisory committee chairman node *Cloud*.
4. Node selection stage. $Cloud_l$, as the aggregation server for federated learning global models, selects a global model *W* based on task requests and sends it to the management node RS of the vehicle cluster. Different aggregation accuracy requirements are assigned based on the aggregation score *W*(*RS*) of each management node. Then, based on the node selection algorithm, $Cloud_l$ selects vehicle $v_p \in V$ with high computing and communication capabilities on different reputation $R_2$ partitions. The management node $RS_k$ of each vehicle cluster assigns the initialization parameter $w_{ini}$ of the local model to the participating vehicles $v_p \subset V_p$ locally.
5. Model training phase. The vehicle, RSU, and cloud collaborate to run federated learning to complete the task request *Req*. The trained global model *M* is returned to the requester as a request response and cached locally by the requester’s management node for future requests. The participating vehicles in the vehicle cluster, $V_p \subset V$, perform local model iteration through DAG consensus locally. The management node RS acts as a local server to manage local model updates and collaborates with other management nodes to send local models to the cloud. $Cloud_l$ synchronously updates the global model based on Eq. (20).
6. Shared evaluation and new block generation stage. The management node $RS_k$ verifies and stores node information in the local DAG network, and calculates the training contribution reputation $R_3$ of the vehicle *i* according to Eq. (15). Shared events between participating vehicles and management nodes, as well as between management nodes and cloud regulatory committee nodes, are generated as transactions. All records are collected by the current leader into blocks, generating block headers and volumes, and broadcasted in the vehicle blockchain.
7. Reaching consensus and going live on the blockchain stage. The consensus process of new blocks in the vehicle blockchain is executed by all consensus nodes. New blocks require all regulatory committee nodes and more than half of the management committee nodes (excluding leaders) to verify signatures and update new blocks in the local vehicle blockchain, which cannot be tampered with. The cloud service providers certified by the regulatory committee as authoritative institutions do not participate in the competition for consensus leaders. The management committee nodes evaluate the competition leaders based on reputation and performance, but new blocks require verification signatures from all regulatory committees and more than half of the management committee nodes. This ensures the security of new blocks while improving consensus efficiency. The specific consensus process can be found in Section 3.3.

## Performance evaluation

### Effectiveness of the node selection mechanism

To evaluate the performance of the proposed algorithm in different complex environments, we conducted experiments on the well-known Apollo trajectory dataset^38^. This dataset covers various lighting conditions and traffic flow densities, integrating complex traffic systems composed of motor vehicles, non-motor vehicles, and pedestrians.

**Fig. 5:**
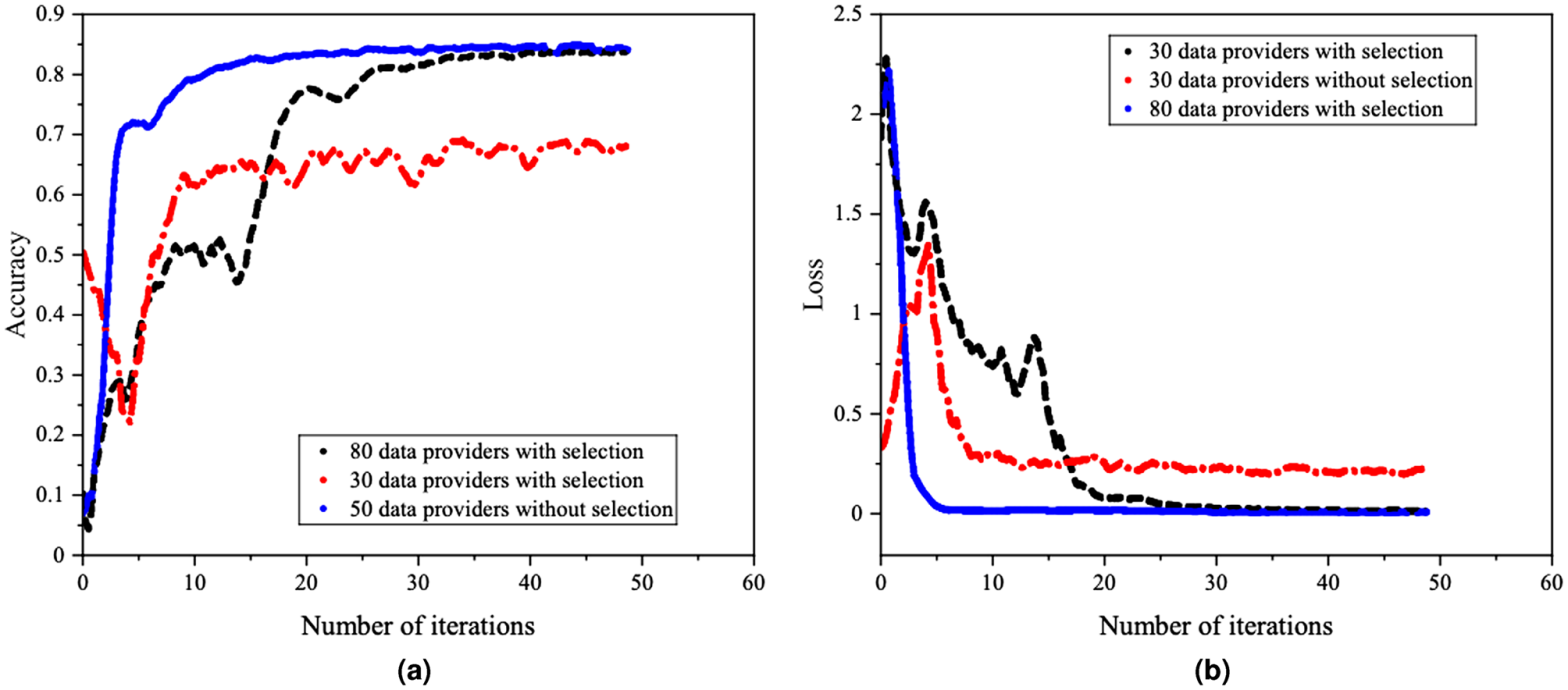
Effectiveness of the node selection mechanism: (**a**) accuracy and (**b**) loss with various numbers of data providers.

To validate the effectiveness of the proposed node-selection mechanism in realistic network settings, we compare model accuracy and loss under different data-provider scales for two settings: with node selection and without node selection, as shown in Fig. 5a and b.

- **Node selection improves accuracy:** Fig. 5acompares model accuracy for 30, 50, and 80 data providers under two settings (with and without node selection). With node selection enabled, increasing providers from 30 to 80 improves the final accuracy, indicating stronger feature extraction capability. In particular, the final accuracy of the 50-provider and 80-provider settings is both above 0.8. This suggests that after filtering low-quality data, adding more providers improves model performance. By contrast, without an effective selection mechanism, simply increasing the number of nodes may introduce additional noise and does not necessarily improve performance.
- **Impact on convergence speed:** The setting without node selection includes noisy data to simulate malicious-node participation in real deployments. As shown in Fig. 5b, noisy data severely impairs convergence. Without node selection (red curve, 30 providers), loss remains above 0.2 even after more than 40 iterations. Under the same data scale but with node selection (black curve), loss drops close to zero at about 25 iterations despite early-stage fluctuations. This indicates that the proposed selection mechanism can effectively filter noisy/malicious participants during iterative training.
- **Adaptive behavior of node selection:** In both Fig. 5a(accuracy) and Fig. 5b(loss), the curves under node selection show stronger oscillations within the first 15 iterations, especially for the black dashed curves. This reflects an intensive early-stage screening process, where node weights are dynamically adjusted and outliers are removed. After this screening phase, the curves become smoother and more stable, demonstrating good adaptability of the selection mechanism.

### System robustness and security

**Fig. 6:**
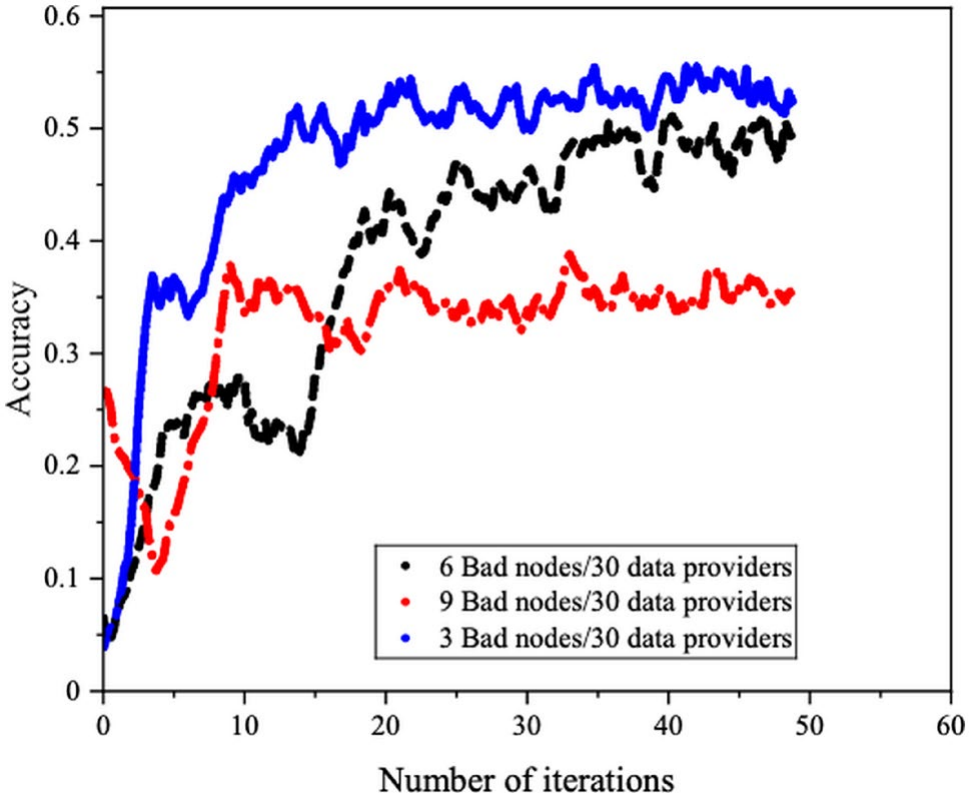
The accuracy in various numbers of bad nodes.

Figure 6 presents model accuracy under poisoning attacks with different numbers of malicious nodes in the same federated-learning setting (30 data providers). It should be noted that this figure reports performance under a challenging robustness setting rather than under a standard clean setting. We evaluate three attack levels: low (3 malicious nodes), medium (6 malicious nodes), and high (9 malicious nodes). The blue curve (low attack level) achieves the highest accuracy (about 0.55), rises rapidly in the first 10 iterations, and then stabilizes. With increased attack intensity, the black curve (medium level) shows larger early-stage oscillations but still converges to about 0.50, indicating good fault tolerance. Under high-intensity attack (red curve), final accuracy drops to about 0.35, but the model does not collapse and remains convergent, demonstrating strong robustness even when malicious nodes account for a relatively high proportion of participants.

### Performance benchmark comparison and efficiency analysis

**Fig. 7:**
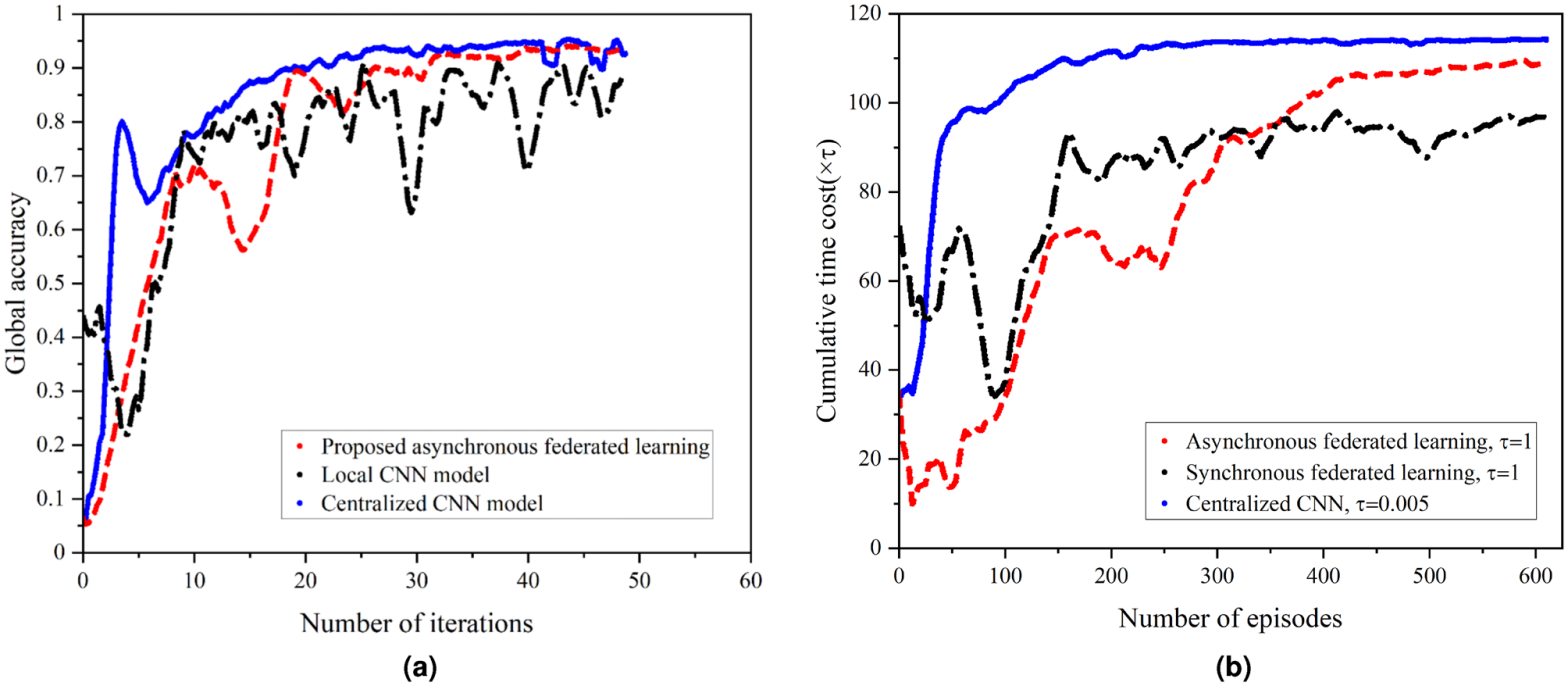
Performance benchmark comparison: (**a**) global accuracy and (**b**) cumulative time cost.

We evaluate asynchronous federated learning from two perspectives, model performance and training efficiency, as shown in Fig. 7aand Fig. 7b.

- **Global accuracy and convergence stability:** Fig. 7a reports 50 training iterations for three models: asynchronous federated learning (our method), a local CNN model, and a centralized CNN model. The centralized CNN (blue) rises rapidly in the first 5–10 iterations, converges at about iteration 15, and stabilizes near 0.93, serving as an upper-bound reference. The local CNN (red) shows strong oscillations and lower final accuracy (about 0.80), reflecting instability caused by local data limitations. Our asynchronous federated learning model (black) is slightly lower than the centralized baseline in the first few iterations, surpasses the local CNN after about iteration 15, and reaches around 0.90 with much smaller oscillations than the local CNN. These results indicate that asynchronous federated learning preserves privacy while achieving robustness close to centralized training.
- **Time cost and communication efficiency:** Fig. 7b compares cumulative time cost over 600 episodes for asynchronous federated learning, synchronous federated learning, and a centralized CNN model. Both federated settings (red and black) show substantially lower cumulative time cost than the centralized model (blue), confirming the communication-efficiency advantage of federated learning. Comparing asynchronous and synchronous federated learning, the asynchronous curve stays below the synchronous curve during the first 300 episodes and starts from a lower latency level. Although the asynchronous curve becomes slightly higher later, its overall cumulative time cost remains lower. In addition, the asynchronous curve is smoother, while the synchronous curve fluctuates more strongly, indicating better stability for asynchronous federated learning in complex network conditions.

### Feasibility, scalability, and comparative performance analysis

**Fig. 8:**
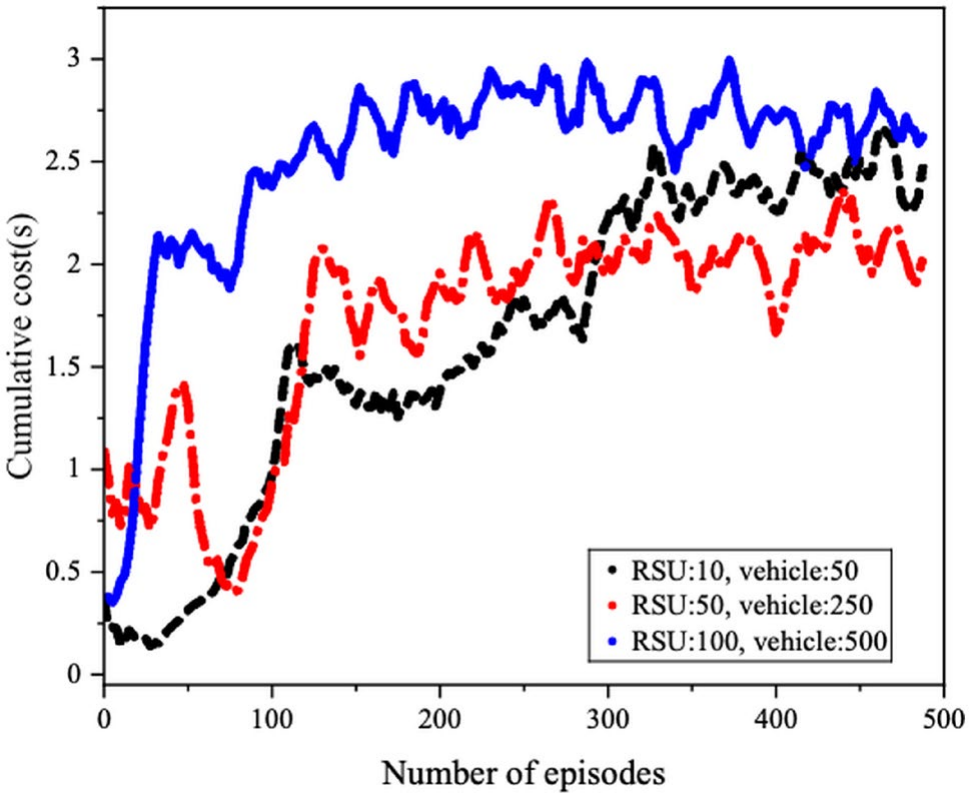
Cumulative cost of the proposed blockchain scheme.

**Fig. 9:**
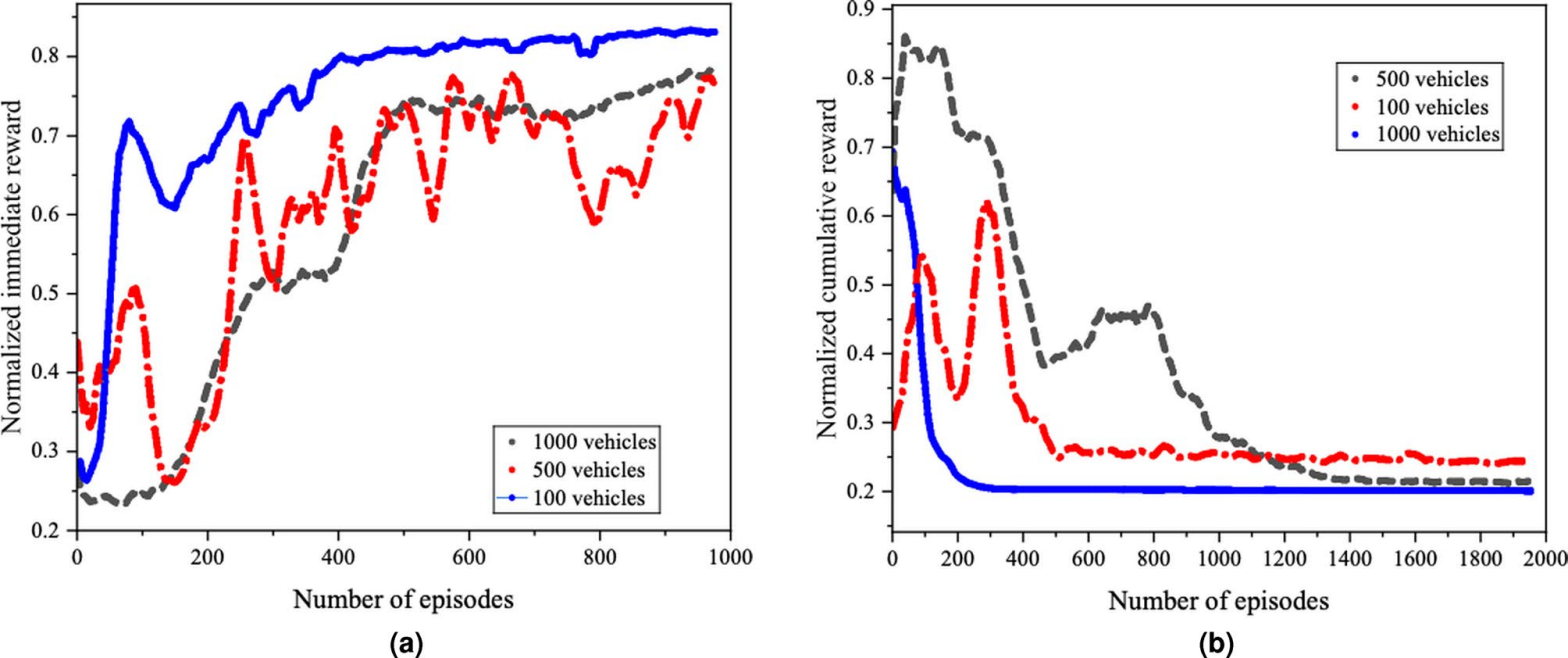
Reward dynamics of DDPG-based selection: (**a**) immediate reward and (**b**) cumulative reward with varying number of vehicles.

To better approximate real-world networking conditions and evaluate the proposed method comprehensively, we consider three scales: small (RSU: 10, vehicles: 50), medium (RSU: 50, vehicles: 250), and large (RSU: 100, vehicles: 500). Fig. 8 shows cumulative cost under these settings. Although the number of vehicles increases tenfold from 50 to 500, cumulative cost rises only modestly (from about 2.5 to about 2.75), indicating good scalability and suitability for high-concurrency IoV environments. Notably, after about 300 episodes, the 50-vehicle curve exceeds the 250-vehicle curve, suggesting that in sparse scenarios consensus may slow down because fewer nearby nodes are available for timely verification and relay.

Figure 9a and b further evaluate the reward dynamics of the DDPG-based selection algorithm across different vehicle scales. Fig. 9a reports immediate reward, whereas Fig. 9b reports cumulative reward, reflecting short-term and long-term adaptation, respectively. In the early stage, the 100-vehicle setting increases fastest, followed by the 500-vehicle setting, while the 1000-vehicle setting grows more gradually because of its larger and more complex state space. In later stages, the immediate reward under 1000 vehicles remains lower than that under 100 vehicles, which is consistent with practical large-scale deployment where the achievable upper bound is lower. From Fig. 9b, convergence occurs at around 200 episodes (100 vehicles), 500 episodes (500 vehicles), and 1300 episodes (1000 vehicles). After convergence, the large-scale setting exhibits low variance, indicating stable policy learning. Overall, these results support the feasibility and scalability of the proposed algorithm across small, medium, and large IoV settings.

Building on this feasibility and scalability analysis, we further compare the proposed framework with state-of-the-art baselines to evaluate its performance in complex environments.

**Fig. 10:**
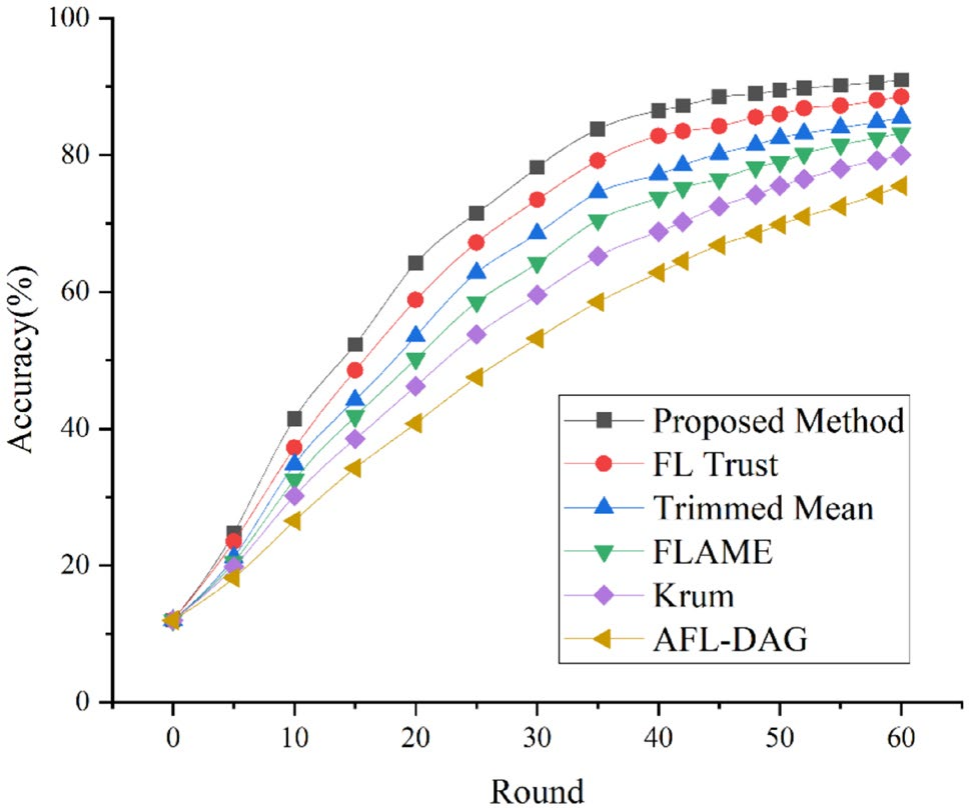
Comparison with State-of-the-Art Federated Learning Baselines.

**Table 4:** Performance comparison of different federated learning methods.

Method	Accuracy (%)	Convergence Rounds (to 85%)	Cumulative Time Cost (s)	Robustness Degradation (%)	Communication Overhead (MB)
Proposed Method	91.0	31	245	3.0	128
FLTrust	88.5	43	395	5.5	152
Trimmed Mean	85.5	51	365	8.5	135
FLAME	83.2	56	390	10.8	140
Krum	80.0	60	378	14.0	118
AFL-DAG	75.5	>60	515	18.5	148

To evaluate the performance of the Vehicle-Road-Cloud-Chain (VRCC) framework in complex environments such as differential privacy noise, Non-IID data, and Byzantine attacks, this section introduces five baseline methods for comparison: Krum^9^, FLAME^16^, Trimmed Mean^10^, FLTrust^28^, and AFL-DAG^35^. The experiment conducts an analysis from five dimensions: global accuracy, convergence speed, system robustness, time cost, and communication overhead. The results are shown in Fig. 10 and Table 4.

In terms of global accuracy and convergence efficiency (Fig. 10), the initial performance of all methods is similar; subsequently, the accuracy of the proposed method increases relatively faster, ultimately reaching 91.0%. Combined with Table 4, the proposed method requires 31 rounds to reach an 85% accuracy, which is less than the 43 rounds of FLTrust, whereas AFL-DAG fails to meet this standard within 60 rounds. This is primarily due to the fact that in the early stages of training, the proposed mechanism filters reliable nodes through reputation evaluation, reducing the impact of low-quality data, thereby promoting the accumulation of high-quality model gradients.

Regarding the defense against malicious nodes and the comprehensive system operating overhead, the accuracy of the proposed method only drops by 3.0% under Byzantine attacks (Table 4), and its interference resistance is superior to baseline methods such as FLTrust (5.5%). This is largely attributed to the three-tier reputation evaluation mechanism constructed in this paper—it integrates historical performance and MMD-based Bayesian inference, and substitutes the traditional Mean Absolute Error (MAE) with Cosine Similarity in the model contribution evaluation ($R_3$). This effectively reduces the misjudgment and over-clipping of useful gradients in highly Non-IID data and privacy noise environments. Simultaneously, the DAG-based asynchronous consensus layer mitigates the delay caused by waiting for “stragglers” in traditional synchronous federated learning, reducing the cumulative time cost of the proposed method to 245 seconds, which is lower than that of AFL-DAG (515 seconds) and FLTrust (395 seconds). In terms of communication overhead, the proposed method remains controlled at 128MB even with the introduction of multi-layer reputation interactions. Although slightly higher than the Krum algorithm (118MB), which comes at the cost of significantly sacrificing accuracy, overall, this framework achieves a better balance among security, convergence efficiency, and system resource overhead in the Internet of Vehicles environment.

## Conclusion

In this article, we address the main challenges in the complex IoV environment: stringent data privacy and security requirements, dynamic and heterogeneous network conditions, and scarce communication resources under high-density vehicle scenarios, and propose the Vehicle-Road-Cloud-Chain (VRCC) framework that integrates a DAG-based distributed asynchronous architecture with a causally transparent three-layer reputation mechanism. The experimental results are validated from multiple aspects, demonstrating the effectiveness, stability, and superiority of the proposed method in mitigating Byzantine attacks and extreme Non-IID conditions. In terms of system cost, the results show that even when the number of vehicles increases by a factor of 10 in large-scale vehicle scenarios, the cost only rises from approximately 2.5 to approximately 2.75, without exhibiting exponential growth but instead maintaining a high degree of controllability, which fully proves the excellent scalability of the proposed DAG-based scheme and its ability to adapt to high-concurrency IoV environments.

The combination of DAG-based blockchain and federated learning offers a feasible approach to secure data sharing in IoV. However, in practical IoV scenarios, how to truly achieve efficient privacy protection using blockchain still remains an open question, and more real-world security threats need to be confronted to gradually work out more practical solutions. Moreover, whether the shared data models are actually useful and whether they can function independently of specific tasks and algorithms is equally tricky — ultimately, smarter methods that provide causal transparency and interpretability are needed to make the data itself more valuable. Furthermore, the computation and communication resources on vehicle terminals are inherently limited, and how to further improve the efficiency of data sharing under such constraints remains an unavoidable practical challenge.

## Data Availability

The datasets generated and analysed during the current study are available in the [GRIP++: Enhanced Graph-based Interaction-aware Trajectory Prediction for Autonomous Driving] repository, [https://apolloscape.auto/trajectory.html#]^38^
